# Genome-wide analysis of the aquaporin genes in melon (*Cucumis melo L.*)

**DOI:** 10.1038/s41598-020-79250-w

**Published:** 2020-12-17

**Authors:** Alvaro Lopez-Zaplana, Juan Nicolas-Espinosa, Micaela Carvajal, Gloria Bárzana

**Affiliations:** Aquaporins Group, Plant Nutrition Department, Centro de Edafología Y Biología Aplicada del Segura (CEBAS-CSIC), Campus Universitario de Espinardo, Edificio 25, 30100 Murcia, Spain

**Keywords:** Computational biology and bioinformatics, Genetics, Molecular biology, Plant sciences

## Abstract

Melon (*Cucumis melo* L.) is a very important crop throughout the world and has great economic importance, in part due to its nutritional properties. It prefers well-drained soil with low acidity and has a strong demand for water during fruit set. Therefore, a correct water balance—involving aquaporins—is necessary to maintain the plants in optimal condition. This manuscript describes the identification and comparative analysis of the complete set of aquaporins in melon. 31 aquaporin genes were identified, classified and analysed according to the evolutionary relationship of melon with related plant species. The individual role of each aquaporin in the transport of water, ions and small molecules was discussed. Finally, qPCR revealed that almost all melon aquaporins in roots and leaves were constitutively expressed. However, the high variations in expression among them point to different roles in water and solute transport, providing important features as that CmPIP1;1 is the predominant isoform and CmTIP1;1 is revealed as the most important osmoregulator in the tonoplast under optimal conditions. The results of this work pointing to the physiological importance of each individual aquaporin of melon opening a field of knowledge that deserves to be investigated.

## Introduction

Aquaporins are highly conserved transmembrane proteins, present in all domains of life, whose main function is the selective, bidirectional and passive transport of water and some small neutral solutes and ions^1^. Plant aquaporins are classified within the superfamily of Major Intrinsic Proteins (MIPs). The classification of the aquaporins into five subfamilies provide the PIPs, (plasma membrane intrinsic Proteins), the TIPs (tonoplast intrinsic proteins), the NIPs (nodulin26-like intrinsic proteins), SIPs (small basic intrinsic proteins)^2^, XIPs (X-intrinsic proteins), depending on their sequence homology and subcellular localisation. Aquaporins are formed by four monomers, each monomer constituting a functional pore, that together constitute stable tetramers formed by interaction of the monomers with each other through neighbouring membrane-spanning α-helices, via hydrophobic interactions and hydrogen bonds^3^. This close association forms an additional fifth, central pore with suggested functions in gases and ion transport^4,5^. Each monomer is configured by six membrane-spanning α-helices (H1–H3 and H4–H6) and two re-entrant short α-helices (HB and HE) with five interconnecting loops (LA-LE) that collectively form a right-handed α-helical bundle, configuring the typical hourglass morphology with two opposite vestibules. The N- and C-termini are both located on the cytoplasmic side of the membrane^6^.

The specificity of the solute transport through the different aquaporin homologues is mainly controlled by steric occlusion provided by the specific residues that constitute each monomer; among these, the two main constrictions of the pore channel appear to have a determining role. The first constriction is formed at the center of the “hourglass” pore by the opposite juxtaposition of two Asn-Pro-Ala (NPA) motifs situated at the end of the two re-entrant helices, in loops B and E^6^. NPA motifs have a critical function in proton exclusion and as a size barrier. The second constriction, known as the aromatic/arginine (ar/R) selectivity filter, is formed at the extracellular vestibule by four residues, one each in helices 2 and 5 (H2, H5) and two in Loop E (LE1 and LE2), and is the narrowest part of the pore in most of the aquaporin homologues^7^ that give rise to the possibility of transporting different specific molecules while serving as an exclusion barrier for others^8^ (for a review, see Luang and Hrmova, 2017)^3^. However, in addition to these two well-known filters, other amino acid residues have been shown to be important in the discrimination between the transport of molecules, the Froger’s positions (FPs)^9^ located in Loop C (P1), Loop E (P2–P3) and transmembrane helix 6 (P4–P5). These residues were described in the first years of aquaporins discovery, but they have been little studied^10–12^.

In this way, in addition to water, plant aquaporins have been investigated for their capacity to transport a wide range of solutes such as carbon dioxide (CO_2_), hydrogen peroxide (H_2_O_2_), short polyols like glycerol (Gly), NH_3_, urea, boric acid (boron, B), silicic acid (silicon, Si), arsenic (As), antimonite (Sb) and some other compounds like germanic acid, selenic acid, lactic acid, formamide and acetamide (for a detailed bibliography see Luang and Hrmova, 2017)^3^. This reveals the great influence that aquaporins can have on the control of growth, nutrition, osmoregulation, signaling, ion homeostasis and defense against different stresses^13–15^.

Melon (*Cucumis melo* L.) is a eudicot diploid species (2n = 24) and one of the most important cucurbits. The great importance of melon is due not only to its great economic importance but also to its nutritional properties as the presence of health-promoters including β-carotenes, folic acid, phenolic acids, vitamins A and C and minerals^16,17^, being of special interest in nutrition and human healthcare^18^. The crop highly demand an appropriate water balance^19^ related with its capacity to adapt quickly under abiotic stress conditions. This suggests strong control of water and nutrients transport in membranes, pointing to aquaporins as one of the most interesting targets in these plants.

Therefore, as each aquaporin is structurally unique and that simple variations of specific residues can produce an altered solute selectivity, the aim of this study was to identify, compare and analyse the complete set of aquaporins in melon. The comparative analysis of aquaporins was performed to understand the evolutionary relationship of melon with related plant species, to predict their possible roles in the transport of other compounds apart from water and to determine their constitutive expression (determining structural importance of NPA motifs, the ar/R region and FPs). Analysis by qPCR was carried out in roots and leaves after the design of primers and a comparison with RNA-seq results was done with existing databases.

## Material and methods

### Identification of putative *C. melo* aquaporins (CmAQPs)

The complete set of aquaporin protein sequences of watermelon (*Citrullus lanatus* L.)^20^*,* identified by Zhou et al., 2019, was used as a template in the PSI-Blast tool (https://blast.ncbi.nlm.nih.gov/Blast.cgi) with default parameters and the three-iterations method. This was performed against the *C. melo* database and using the non-redundant protein sequences (nr) database.

All the sequence-related information was retrieved from the National Centre of Biotechnology Information (NCBI) database (https://www.ncbi.nlm.nih.gov/bioproject/PRJNA246165)^21^ and compared with the corresponding sequences found in the Cucurbit database (CuGenDB) (http://cucurbitgenomics.org/organism/18) collection. Exon–intron analysis of sequences were performed using Microsoft Office package (2016) software (Supplementary Figure S1).

### Protein properties, sequence analysis and phylogenetic studies

Protein features such as the isoelectric point (Pi) and molecular weight (Mw) were calculated with Expasy’s ProtParam tool (https://web.expasy.org/protparam/). The transmembrane domains and number of transmembrane helices were elucidated using the TMHMM server (http://www.cbs.dtu.dk/services/TMHMM/)^22^. Motifs locations was performed by MEME web server (http://meme-suite.org/tools/meme). The subcellular location was predicted with two different prediction softwares: Plant-mPLoc (http://www.csbio.sjtu.edu.cn/bioinf/plant-multi/) and WoLF PSORT (https://www.genscript.com/wolf-psort.html), using the default parameters. Protein tertiary structures were predicted with the PSIPRED server^23^.

Furthermore, the important residues involved in the specific transport functions of the aquaporins were identified, first, with alignments of each aquaporin family (PIPs, TIPs, NIPs, SIPs and XIP) performed in the Mega X software with the MUSCLE algorithm^24^. Second, we used the already-described *Spinacia oleracea* PIP1 protein (accession number AAA99274.2) as a pattern to locate all the functional residues—like the NPA motifs (loop B and loop E, respectively), the ar/R filters (H2, H5, LE1, LE2)^6,25,26^ and the FPs (P1-P5) described by Froger et al. (1998)^9^. The prediction of the transport of substances other than water was obtained by comparison with orthologues and their known functions in *Arabidopsis thaliana* L., rice (*Oryza sativa* L.) and maize (*Zea mays* L.)*.* Their functions as transporters are related to the same structural positions^11,12^. In addition to this, comparisons of homologues^10,11^, mutational studies^12,26–28^, pore structure analyses^29,30^ and experimental studies^12,13,26–28,31–46^ have been taken into account, leading us to suggest the transport possibilities for each aquaporin of *C. melo*.

**Figure 1:**
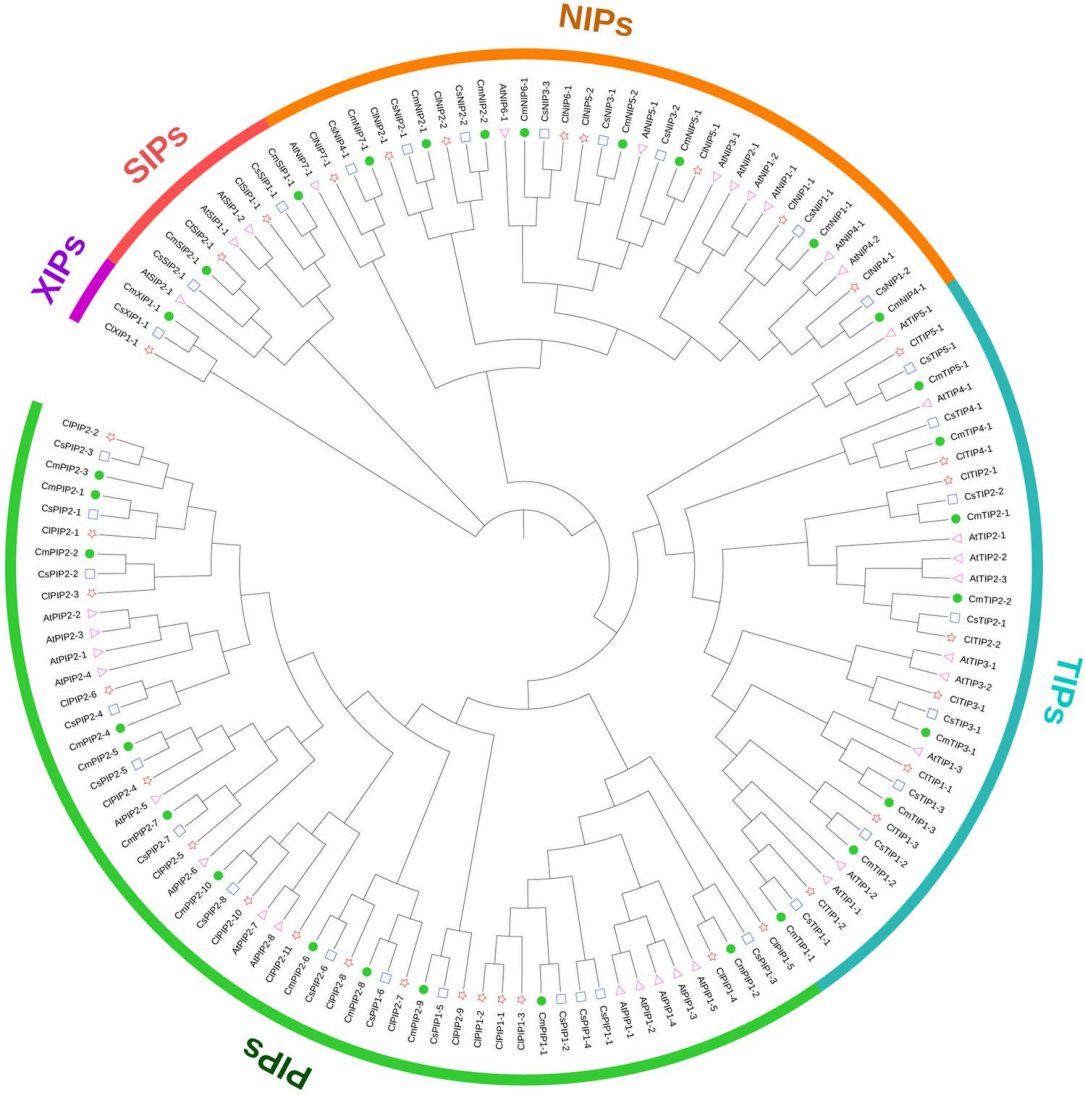
Phylogenetic analysis of aquaporin family proteins of *C. melo* (filled circles), *A. thaliana* (triangles), *C. lanatus* (stars) and *C. sativus* (squares). We used MUSLE to align the protein sequences and the NJ method (with 1000 bootstrap replications) to build the tree, all with MEGA X. Phylogenetic tree design has been done with the online tool “Interactive Tree Of Life” (iTOL; https://itol.embl.de/). The different MIP sub-families are highlighted as: PIPs (green; PIP1s light green and PIP2s dark green), TIPs (sky blue), NIPs (orange), SIPs (pink) and XIPs (purple). The abbreviation of the species is as follows: Cm (*C. melo)*, At (*A. thaliana*), Cl (*C. lanatus*), Cs (*C. sativus*). The meanings of the suffixes in the *C. sativus* names are: lk: like, pb: probable, pd: predicted.

For the phylogenetic analysis and tree construction, *C. melo* aquaporins protein sequences were aligned with all the characterised aquaporins from *C. lanatus*^20^, *A. thaliana*^2^ and *Cucumis sativus* L.^47^ (Fig. 1). Mega X was employed to build the phylogenetic tree. We aligned all the sequences with the MUSCLE method and then used a Neighbour Joining (NJ) algorithm employing 1000 bootstrap replicates, a Poisson model and pairwise deletion. The same analysis and tree construction were performed for the *C. melo*, *A. thaliana*, *Z. mays*^48^ and *O. sativa*^49^ phylogenetic tree (Supplementary Figure S2).

### Plant material and growth conditions

Seeds of melon, *C. melo* var. Grand Riado, supplied by SAKATA SEED IBERICA S.L.U, were hydrated with deionised water and aerated for 16 h. After this, they were germinated in vermiculite, in the dark at 28 °C, for 2 d. They were then transferred to a controlled-environment chamber having a 16-h light and 8-h dark cycle with temperatures of 25 and 20 °C and relative humidity of 60 and 80%, respectively. Photosynthetically active radiation (PAR) of 400 µmol m^−2^ s^−1^ was provided by a combination of fluorescent tubes (PHILIPS TLD 36 W/83, Jena, Germany and Sylvania F36 W/GRO, Manchester, NH, USA) and metal halide lamps (OSRAM HQI, T 400 W, Berlin, Germany). After 3 d, once the seeds germinated, 16 seedlings were placed in 15-L containers, 4 seedlings per container, with continuously-aerated Hoagland nutrient solution^50^. The solution was replaced completely every week. After a month of growth, 6 plants were harvested separating roots and leaves from the rest of the plant, instantly frozen in liquid nitrogen and stored at − 80 ºC. The experimental design was completed randomized design (CRD).

### RNA extraction and retro transcription (RT)

Total RNA was extracted using the RNeasy Plant Mini Kit (QIAGEN, Hilden, Germany), according to the manufacturer’s protocol. The quantity and purity of RNA was measured with a Nanodrop 1000 spectrophotometer (Thermo Fisher Scientific, USA). Integrity of the RNA was measured by electrophoresis in agarose gel. Contaminating DNA was removed using RNase-free DNase solution, (Thermo Fisher Scientific) according to the manufacturer’s instructions. The RNA extracted was stored at − 80 °C until use. The absence of DNA contamination was checked in all samples by polymerase chain reaction (PCR) using recombinant Taq DNA Polymerase (Thermo Fisher Scientific) with aliquots of the same RNA that had been subjected to the DNase treatment but not to the reverse-transcription step according to the manufacturer’s protocol. The High-Capacity cDNA Reverse Transcription Kit (Thermo Fisher Scientific) was used to synthesise cDNA from 2 µg of total RNA, according to the manufacturer’s protocol.

### Primers design

The primer sets used to amplify each aquaporin gene were specifically designed in the 3′ or 5′ non-coding region of each gene, in order to avoid the non-specific amplification of other aquaporin genes, since in the coding region the homology between their sequences is very high^51^. For this same reason, the primer design was carried out manually, meeting the specific requirements imposed by the high homology of the sequences used and the technique used for the analysis. Virtual analysis of melting temperature, primer hairpins, self-dimers, hetero-dimers and individual and total ΔG was performed with PCR Primer Stats (https://www.bioinformatics.org/sms2/pcr_primer_st) and IDT Oligo Analyzer Tools (https://eu.idtdna.com/calc/analyzer/). The ΔG accepted for dimer analysis was always less than − 6.5 kcal/mol (Table 1). The specificity of the amplicons was checked using the virtual nucleotide basic local alignment search tool (NCBI nucleotide BLAST: https://blast.ncbi.nlm.nih.gov/Blast.cgi) and then physically, with the total DNA extracted from 50 mg of frozen sample (DNeasy Plant Pro Kit. QIAGEN, Hilden, Germany) according to the manufacturer’s protocol, by standard PCR using Taq DNA Polymerase, recombinant (Thermo Scientific). The efficiency of the primer sets was evaluated with the software QuantStudio 5 (QuantStudio Design and Analysis Software version 1.4.0.0), by analysing the threshold cycle (Ct)/fluorescence ratio at six independent points of PCR curves^52^, giving values between 95 and 100% (Table 1). Five housekeeping primers—*CmACT* (gi: MU51303), *CmADP* (gi: MU47713), *CmGAPC2* (gi: MU54550), *CmRAM* (gi: MU45556) and *CmRLP* (gi: MU45916)—for *C. melo*, selected according to Kong et al. (2014)^53^, were checked in each cDNA used in the quantitative PCR quantification (qPCR) and were measured using a Visual basic application for Excel (GeNorm) that automatically calculates the gene stability^54^. *CmRAN* (encoding the GTP-binding nuclear protein) was then selected as the reference gene for the standardisation of each sample. The sequences and features of the primers used for the 31 melon aquaporin genes and one constitutively-expressed gene are shown in Table 1.

**Table 1:** Primer used for measurement of C*. melo* (Cm) aquaporins expression by RT-qPCR.

Gene name	Primer sequence 5′– > 3’ forward/reverse primer)	Product size (bp)	ΔG Primer	ΔG Self-dimer	ΔG hetero-dimer	E (%)	Tª melting	Tª annealing	DNA dilution
*CmRAN* ^#^	F:TGCACCCTTTGGACTTCTTC R:GATGTAGTAGCCATCCCGTAAAC	104	–	–	–	98.7	–	–	–
*CmPIP1;1*	F: GCTTCTTCAATCAATCACAGC R: ACCATTACATAGCTTCATAGCC	181	− 37.26 − 38.53	− 4.74 − 6.34	− 4.74	99.7	75.81	60 °C	1:10
*CmPIP1;2*	F: CATTCTTCCCCAAAAGCAAA R: AGTTTTCAGAAAGGCAGCCA	179	− 39.64 − 38.89	− 3.54 − 6.21	− 5.49	98.81	76.17	60 °C	1:10
*CmPIP2;1*	F: TTAAGCCTAATAGTTGTGTGC R: AAAAGAGAGCAAAAACCACG	147	− 36.17 − 39.91	− 4.85 − 3.61	− 5.09	100.36	75.16	60 °C	1:2
*CmPIP2;2*	F: GGATCTAAAATGTGTGATTAGG R: ATAAAACCTTCAAAATATGTGC	158	− 36.73 − 37.88	− 4.62 − 3.91	− 4.67	100.15	73.91	57 °C	1:10
*CmPIP2;3*	F: ATCTCTAACATCCATCACTCC R: TAGTTAGCTTGTGGTGGTCG	94	− 34.68 − 36.3	− 1.47 − 6.34	− 5.85	99.98	76.96	60 °C	1:5
*CmPIP2;4*	F: CTCTCGTCTGCTTTGGTCC R: ATACATAGGATGAGAATGAGC	158	− 36.06 − 34.27	− 3.61 − 3.43	− 4.75	99.13	75.14	60 °C	1:5
*CmPIP2;5*	F: TTCCTTTTGCTATTGGAATGG R: TTGCCTGTTACATTACTAGG	89	− 42.08 − 36.1	− 6.59 − 4.67	− 5.37	100.32	71.69	57 °C	1:5
*CmPIP2;6*	F: TCTGCTCTATAAATCTTATCCC R: TAACACACTTCATTAGTTCAGC	144	− 37.07 − 35.44	− 4.38 − 3.29	− 4.74	99.43	74.25	57 °C	1:2
*CmPIP2;7*	F: CCATTCCATAAGCAAAAGACT R: GAACATTAGTAAGCCAAGTGG	177	− 38.06 − 36.71	− 3.14 − 5.02	− 5.02	96.27	72.00	60 °C	1:2
*CmPIP2;8*	F: ACAAAACCAAAGAAGTGTTCG R: GCAGGATCTCAGTGAATGTG	158	− 37.87 − 34.65	− 3.61 − 4.62	− 3.52	99.72	76.51	60 °C	1:5
*CmPIP2;9*	F: CTTCTTCCTTACACTTCATGC R: TTACCCAATTACAAAAGATTGC	119	− 36.09 − 39.48	− 5.38 − 5.37	− 5.12	97.43	73.48	60 °C	1:2
*CmPIP2;10*	F: AAGAAGATGATGGTAGAAGTGG R: CATTCAAAGACAATCCCTTCC	120	− 37.12 − 38.58	− 1.47 − 3.54	− 5.12	99.95	73.21	60 °C	1:10
*CmTIP1;1*	F: CGTCAACTTCTTTGTTCTACGT R: CAATTTAATACGACATCAAAATGG	90	− 38.04 − 41.76	− 6.30 − 5.36	− 5.84	95.41	73.29	60 °C	1:10
*CmTIP1;2*	F: TCAACCACCACCACCACC R: GACACGACCAAACCCATCC	129	− 34.34 − 37.21	Non − 3.61	− 1.57	99.44	75.19	60 °C	1:5
*CmTIP1;3*	F: TCTTGACTTTATTCAGAGACC R: ATTCTCTTCCTGATTCTTAGC	105	− 34.14 − 35.72	− 3.53 − 3.14	− 6.35	99.90	72.66	57 °C	1:2
*CmTIP2;1*	F: TCCCTTTGTAATAAGAGGAGG R: AAGAAGAGAATCCAATGAACC	133	− 37.87 − 36.73	− 4.67 − 1.95	− 4.64	99.64	73.77	60 °C	1:10
*CmTIP2;2*	F: GTGTAAAAAATGAAACCAAAACG R: TTGAGGGAAAACCGAAGAAGG	150	− 41.36 − 41.95	− 1.95 − 3.61	− 3.90	99.82	77.43	60 °C	1:2
*CmTIP3;1*	F: TTTCTGCTCTATATGTTGTAGG R: CTGTATGACATTTATTACCTTC	144	− 35.96 − 34.39	− 3.91 − 3.43	− 4.77	100.3	75.69	57 °C	1:10
*CmTIP4;1*	F: GTCATCATACTTACCATTTGC R: ACTACAAGAAACTGGAAAGG	104	− 34.98 − 34.30	− 3.14 − 1.95	− 5.02	98.37	74.58	60 °C	1:2
*CmTIP5;1*	F: TTTAAGCGTTGGTTTTGTGC R: GATAAAAATTCATGTTAGATACAC	97	− 39.02 − 36.66	− 4.85 − 5.38	− 5.83	99.10	72.01	60 °C	1:2
*CmNIP1;1*	F: CCTTACTTCACATGAAACTAGG R: CAGCCATCAAGAAGTTTGG	99	− 36.74 − 35.63	− 5.47 − 5.02	− 6.83	100.3	73.11	60 °C	1:5
*CmNIP2;1*	F: ATAGTTTGAGTGTTTTAATGAGC R: GGCTACTTCTGATACATTGC	124	− 37.51 − 34.6	− 4.85 − 3.14	− 5.37	100.3	71.99	60 °C	1:10
*CmNIP2;2*	F: GAGAAGAATGAATCTGAAATAGG R: GAAAAGAAGAACCAATTTTATGG	115	− 37.93 − 40.72	− 3.17 − 5.83	− 5.36	99.05	70.03	60 °C	1:5
*CmNIP4;1*	F: AAAGGAAGAACATAAACGATAAC R: ATTGAGTCTCAGAAAGAAAGG	86	− 39.06 − 35.38	− 3.61 − 5.13	− 3.9	99.21	72.27	60 °C	1:5
*CmNIP5;1*	F: AGAATAAAGTTGAGAAGAAAAGG R: GGCAAGTAGAAACAATATAGCA	95	− 38.65 − 37.87	− 1.94 − 3.91	− 3.9	98.87	73.47	60 °C	1:10
*CmNIP5;2*	F: TGATAATGATAGTGGTCGTTG R: TTGACATGAAAGTAAAAGGTCG	75	− 35.08 − 38.59	− 3.61 − 5.38	− 3.61	100.2	72.54	60 °C	1:5
*CmNIP6;1*	F: CCCAGAGAACACTTTGAACC R: ATACACAATGACCAATACTTGC	137	− 36.39 − 36.7	− 1.95 − 3.90	− 3.9	100.2	75.56	60 °C	1:5
*CmNIP7;1*	F: CCCTCTATATTTCCAGTTGC R: AAGTAAGGTTTAATTTGATTACCG	132	− 36.25 − 41.89	− 3.91 − 5.36	− 4.67	100.7	72.46	60 °C	1:2
*CmSIP1;1*	F: GCAGTTATGTTTAGTTTGATTC R: CTAATGTCCAAAGTCTATAAGC	92	− 35.91 − 35.95	− 3.14 − 3.40	− 5.84	100.5	71.54	60 °C	1:2
*CmSIP2;1*	F: CTCTTAATGATTCCAATGTAGTG R: TGACGATGTTGTCGGATTCC	101	− 37.21 − 37.96	− 4.85 − 5.19	− 4.64	98.77	76.71	60 °C	1:2
*CmXIP1;1*	F: TCTTCCTCTTTTCTCTCAAGG R: GCAGTCGCTACTAATTCTGTC	101	− 36.86 − 36.23	− 4.67 − 5.36	− 1.95	NM	71.82	60 °C	1:2

Columns: gene name, primer sequence: up the forward (F) and below the reverse (R), product size (bp), ΔG primer, ΔG self-dimer, ΔG hetero-dimer between primers, % efficiency (E), Tª melting and qPCR conditions (Tª annealing and DNA dilution).

Shortening code: NM, No measurement.

^#^*CmRAN* primers were obtained from Kong et al.^53^.

### Quantitative real-time PCR (RT-qPCR) analyses

To compare the expression of all aquaporin genes, RT-qPCR was carried out. It was performed using 2 μL of 1:2, 1:5 or 1:10 diluted cDNA samples, depending on the gene being analyzed (see Table 1), in 10 μL of reaction medium containing 500 nM gene-specific primers and 5 μL of Power SYBR Green PCR Master Mix (Applied Biosystems by Thermo Fisher Scientific), in a QuantStudio 5 Flex, a Real-Time qPCR system (Applied Biosystems by Thermo Fisher Scientific) following the manufacturer’s instructions. The qPCR program consisted of a 10 min initial denaturation at 95 °C and then amplification in a two-step procedure: 15 s of denaturation at 95 °C and 60 s of annealing and extension at a primer-specific temperature for 40 cycles, followed by a dissociation stage. Data collection was carried out at the end of each round in step 2. These conditions were used for both target and reference genes, and the absence of primer-dimers was checked in controls lacking templates. Real-time PCR analysis was performed on 3–6 independent samples for each treatment (biological replicates) and each sample reaction was carried out in triplicate (technical replicates) in 96-well plates. The transcript levels were calculated using the 2−ΔΔCt method^55^. Negative controls without cDNA were used in all PCR reactions. Finally, the normalised expression levels were rescaled and presented as relative units (ru) with respect to the most highly-expressed aquaporin, *CmTIP1;1*, which was assigned a value of 100. The extremely high expression of *CmTIP1;1* masks the presence of other aquaporins, which is why we separated them into three different groups depending on their relative levels of expression.

### Comparison with previous RNA-seq

Our qPCR analyses were compared with the RNA-seq analyses in databases, namely, the work of Latrasse et al. (2017)^56^. For this, the levels of all RT-qPCRs were relativized to that of the gene that had the highest expression levels, as previously described. The same procedure was followed with all the genes analysed by RNA-seq. In this case, the maximum value was for *PIP1;1* in roots, which received the same fixed value of 100, and the rest were relativized to it. Finally, the values of each aquaporin in the RNA-seq were compared with those obtained from our RT-qPCR. After being relativized, all aquaporins data were separated in groups of similar relative expression, as in the previous case of the qPCR data.

### Data analysis

Statistical analyses were performed using the SPSS 25.0.0.1 software package. For the qPCR analyses, the Student’s t-test was performed. The qPCR and RNA-seq comparisons were analysed using one-way ANOVA, followed by the post hoc Tukey multiple comparison test. Significant differences between the values of all parameters were determined at *P* ≤ 0.05, according to Tukey’s test. The values presented are the means ± SE. To detect outliers in the qPCRs performed, the SPSS 25.0.0.1 software package was used.

### Ethical approval

This article does not contain any studies with human or animal subjects.

### Consent to participate

All the authors referred in this study acknowledge the authorship and agreed with the content.

### Consent of publication

All the authors referred in this study give explicit consent to submit this article and consent to the publication of the data presented here.

## Results

### Genome-wide identification of CmAQP genes

A search of the whole genome for aquaporins proteins revealed 57 matches in the NCBI protein database, 34 corresponding to *C. melo*, while the rest of the non-selected sequences were specific to concrete varieties.

First, we analysed each matched sequence and searched for its accession in the Cucurbit genomics database. During this procedure we found three sequences (MELO3C025166.2, MELO3C000776.2 and MELO3C025165.2) that were not previously located in our first search of the NCBI protein database. After this, we analysed all the sequences, including these new finds, which resulted in the elimination of some sequences based on the following.

The *CmTIP1;3* gene had three transcript variants in the nucleotide database and only one of them had the characteristic NPA motifs when transcribed to protein. So, the other two transcript variants were omitted.

Also, we found a high nucleotide sequence similarity among five sequences, the three new sequences found in the Cucurbit genomics database and two sequences from the 34 found in the NCBI database (XP_008466925, XP_008463222.1). Only XP_008463222.1 (from now on named CmPIP1;1) was a complete protein sequence including the two characteristic NPA motifs. Sequence alignments showed that XP_008466925 was, indeed, a partial sequence of *CmPIP1;1* with 100% sequence identity. In addition, the other three sequences also had a high similarity to *CmPIP1;1* but the alignment seemed to be shifted, so we thought these sequences were from the upstream region of the *CmPIP1;1* gene, integrating only the second NPA motif. To prove this theory, we extracted the sequence downstream and upstream of the *CmPIP1;1* gene in chromosome 10 and then aligned it against these three unknown sequences. As a result, all the sequences had at least 98–99% identity with the sequence including the *CmPIP1;1* gene and the alignments were located upstream of *CmPIP1;1*, containing only the second NPA motif in their sequences, as we thought at the beginning. With these results and due to the lack of the two NPA motifs, we decided to omit the sequences from further analysis. The presence of this type of sequence in the database could be due to a non-curated identification method without experimental evidence.

In all the analyses carried out, a total of 31 putative aquaporin genes were identified (Table 2).

**Table 2:** List of the 31 aquaporins found in *C. melo* (Cm).

Identifiers			Gene features		Protein features					Subcellular location	
Gene name	CCDB^a^	Gene^b^	Chromosome location	Exon no	mRNA length	Protein length	Mw^c^ (kDa)	Isoelectric point	TMH^c^	Plant-mPLoc^d^	WolF-PSORT^d^
*CmPIP1;1*	MELO3C025164.2	103501427	chr10: 9455124 .. 9456876 (−)	3	1202 bp	292 aa	31.47	7.67	5	plas	plas
*CmPIP1;2*	MELO3C005685.2	103482758	chr09: 23466873 .. 23469204 (+)	4	1275 bp	286 aa	30.71	9.13	6	plas	plas
*CmPIP2;1*	MELO3C014240.2	103491188	chr05: 4877174 .. 4878743 (+)	4	1172 bp	284 aa	29.86	7.71	7	plas	plas
*CmPIP2;2*	MELO3C014241.2	103491189	chr05: 4867151 .. 4869395 (−)	4	1239 bp	284 aa	30.21	8.78	6	plas	plas
*CmPIP2;3*	MELO3C014239.2	107990277	chr05: 4880993 .. 4882676 (+)	4	1245 bp	284 aa	29.89	8.22	6	plas	plas
*CmPIP2;4*	MELO3C025772.2	103501948	chr11: 28209027 .. 28210647 (−)	3	1381 bp	283 aa	30.36	7.63	6	plas	plas
*CmPIP2;5*	MELO3C019794.2	103496419	chr03: 23650515 .. 23655241 (+)	4	1390 bp	276 aa	29.37	9.56	6	plas	plas
*CmPIP2;6*	MELO3C014238.2	103491187	chr05: 4886375 .. 4887984 (+)	4	1178 bp	279 aa	30.02	8.58	6	plas	plas
*CmPIP2;7*	MELO3C012429.2	103489467	chr10: 537492 .. 541727 (−)	4	1116 bp	287 aa	31.24	9.41	6	plas	plas
*CmPIP2;8*	MELO3C009337.2	103486477	chr04: 32797579 .. 32799526 (+)	3	1490 bp	289 aa	31.03	9.10	6	plas	plas
*CmPIP2;9*	MELO3C014244.2	103491191	chr05: 4857028 .. 4859643 (−)	4	1308 bp	278 aa	29.40	8.58	6	plas	plas
*CmPIP2;10*	MELO3C013347.2	103490279	chr01: 16804141 .. 16806721 (−)	4	1199 bp	280 aa	29.91	9.24	6	plas	plas
*CmTIP1;1*	MELO3C024483.2	103500838	chr08: 10141935 .. 10143868 (+)	2	975 bp	250 aa	25.67	5.64	7	tono	plas
*CmTIP1;2*	MELO3C009377.2	103486517	chr04: 32517961 .. 32520018 (+)	2	988 bp	253 aa	26.34	6.03	7	tono	cyto, tono
*CmTIP1;3*	MELO3C025466.2	103501648	chr09: 5838747 .. 5839429 (−)	3	1145 bp	253 aa	26.55	5.53	6	tono	cyto, plas, tono
*CmTIP2;1*	MELO3C024263.2	103500601	chr01: 35574300 .. 35575883 (+)	3	1232 bp	248 aa	25.43	5.66	6	tono	tono
*CmTIP2;2*	MELO3C005526.2	103482603	chr09: 22169680 .. 22172778 (−)	3	1150 bp	250 aa	25.09	5.39	6	tono	tono
*CmTIP3;1*	MELO3C002183.2	103482730	chr12: 25767948 .. 25770390 (−)	3	1137 bp	284 aa	30.08	7.17	5	tono	mito
*CmTIP4;1*	MELO3C011146.2	103488186	chr03: 28321088 .. 28322961 (+)	3	1087 bp	247 aa	25.70	5.91	7	tono	tono
*CmTIP5;1*	MELO3C005441.2	103504693	chr09: 21620302 .. 21621635 (−)	3	957 bp	260 aa	26.86	8.31	6	Plas	chlo
*CmNIP1;1*	MELO3C007188.2	103484424	chr08: 1361212 .. 1363293 (−)	5	1326 bp	276 aa	29.54	9.48	6	plas	plas
*CmNIP2;1*	MELO3C009870.2	103487002	chr04: 28593871 .. 28598610 (−)	5	1360 bp	287 aa	30.41	9.15	6	plas	plas, tono
*CmNIP2;2*	MELO3C009871.2	103487003	chr04: 28560873 .. 28564531 (−)	5	1232 bp	261 aa	27.52	6.29	6	plas	plas
*CmNIP4;1*	MELO3C020281.2	103496839	chr06: 14916752 .. 14920413 (−)	5	1021 bp	269 aa	28.81	7.64	5	plas	tono
*CmNIP5;1*	MELO3C005818.2	103482897	chr09: 24541790 .. 24547172 (−)	4	1794 bp	298 aa	30.83	8.64	5	plas	plas
*CmNIP5;2*	MELO3C005817.2	103482896	chr09: 24536633 .. 24540050 (−)	5	1044 bp	250 aa	26.19	8.62	6	plas	tono
*CmNIP6;1*	MELO3C017831.2	103494651	chr07: 27500120 .. 27503882 (+)	5	2218 bp	304 aa	31.66	7.64	6	plas	tono
*CmNIP7;1*	MELO3C006559.2	103483738	chr06: 4173677 .. 4176268 (−)	5	1123 bp	268 aa	28.52	6.38	6	plas	plas
*CmSIP1;1*	MELO3C008793.2	103485971	chr08: 26514604 .. 26519163 (+)	3	1287 bp	243 aa	25.58	9.55	6	plas	tono
*CmSIP2;1*	MELO3C009719.2	103486855	chr04: 29912868 .. 29916655 (−)	3	1275 bp	316 aa	34.88	9.72	6	plas	plas
*CmXIP1;1*	MELO3C020774.2	103497290	chr11: 4268119 .. 4268934 (+)	2	1148 bp	316 aa	34.21	6.88	7	plas	plas

Columns: Identifiers (Gene name, ID from Cucurbit genomics database and NCBI accession), Gene features (chromosome location and exons number), protein features (mRNA length, protein length, molecular weight, isoelectric point and predicted number of transmembrane domains) and subcellular location prediction by Plant-mPLoc and Wolf-PSORT programs.

^a^ID from Cucurbit genomics database.

^b^NCBI accessions.

^c^Mw: molecular weight in kDa and TMHC: predicted number of transmembrane domains.

^d^Shortening codes from subcellular location in Plant-mPLoc and Wolf-PSORT. Plas: plasma membrane; cyto: cytoplasm; tono: tonoplast; mito: mitochondria; chlo: chloroplast.

### Nomenclature and classification

For the 31 putative aquaporin genes finally selected, their amino acid sequences were aligned for a phylogenetic analysis that divided them into sub-families, to help us to name them correctly. The *C. melo* aquaporins were named relative to their homology and phylogenetic relationships with those of *C. sativus, C. lanatus and A. thaliana* (Fig. 1).

The proteins were grouped into five sub-families consisting of twelve PIPs, nine TIPs, eight NIPs, two SIPs and one XIP (Fig. 1). The PIP sub-family was divided into two groups (PIP1 and PIP2), with two members in the PIP1 group and ten in PIP2. The TIP sub-family was divided into five groups: TIP1, with three members, TIP2, with two members and one member each in groups TIP3, TIP4 and TIP5. The NIPs were divided into NIP1, NIP4 and NIP7, with one member each, and groups NIP2 and NIP5, with two members each. The SIP sub-family was formed by SIP1 and SIP2, with one member each. Lastly, only one sequence represented the XIP group.

### Chromosomal location, protein features and subcellular localisation prediction

**Figure 2:**
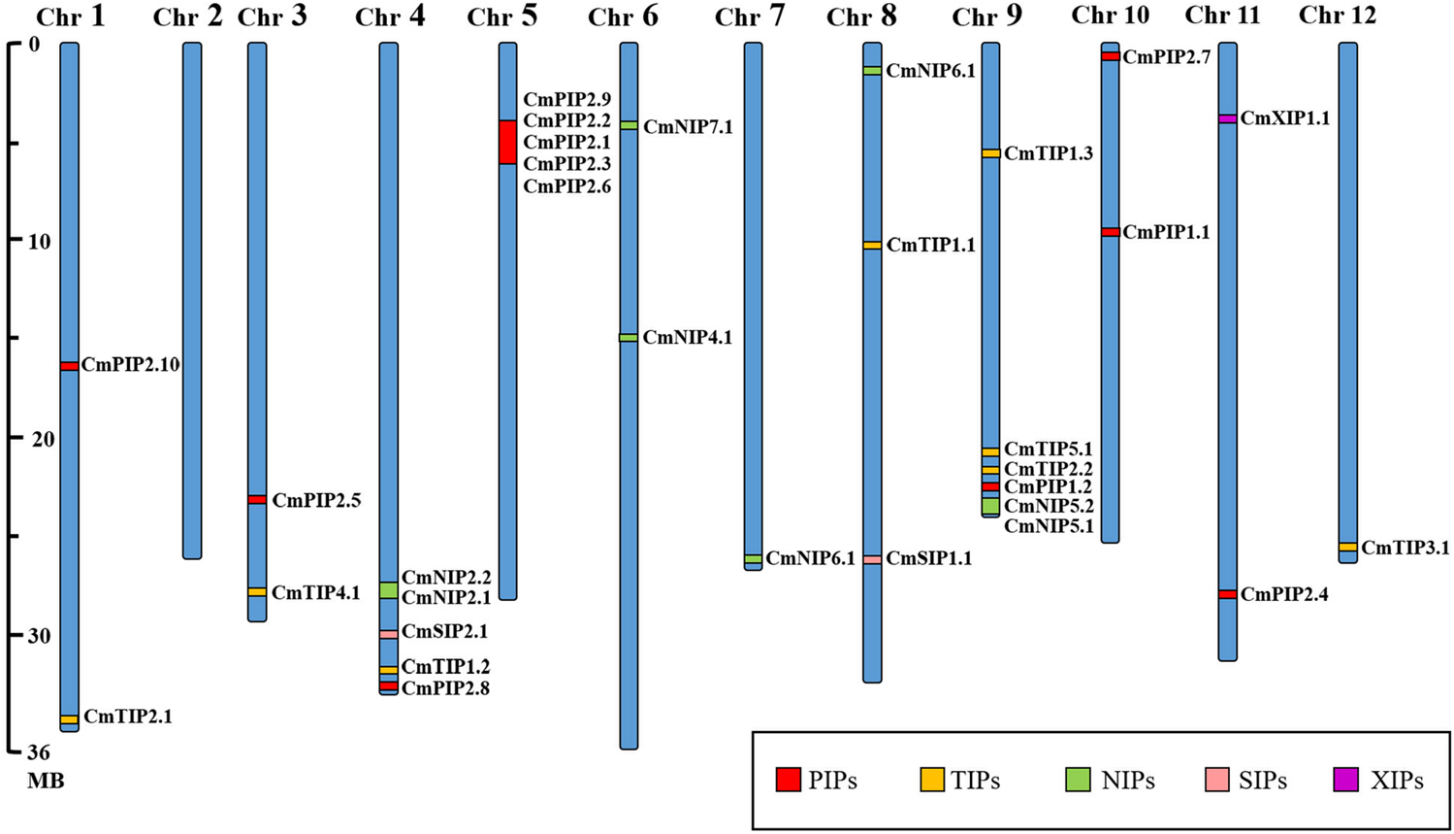
Chromosomal distribution of aquaporin genes in *C. melo*. The different MIP sub-families are distinguished using different-coloured boxes: PIPs (red), TIPs (yellow), NIPs (green), SIPs (pink) and XIPs (purple). The abbreviations are as follows: Cm (*C. melo*), Chr (chromosome). This figure was made with Microsoft Office 2016 package.

These 31 putative aquaporin-encoding genes were located on all chromosomes except chromosome 2, in a non-uniform manner: chromosome 9 incorporated six aquaporins genes, while chromosomes 4 and 5 had five genes each, chromosome 8 had three genes, two genes were identified on each of chromosomes 1, 3, 6, 10 and 11 and chromosomes 7 and 12 possessed only one putative aquaporin gene (Fig. 2). Exon–intron analysis showed a similar distribution on exons structures among each aquaporin family displaying 3–5 exons per gene (Supplementary Figure S1).

The list of identified aquaporins, together with basic statistics on the primary protein sequences, is reported in Table 2. The PIPs protein length ranged from 276 (CmPIP2;5) to 292 residues (CmPIP1;1), with a median of 283 amino acids (aa) per protein, while that of the TIP sub-family ranged from 247 to 284 residues (CmTIP4;1 and CmTIP3;1, respectively), with a median of 255 aa. In the case of the NIPs, the length varied between 250 (CmNIP5;2) and 305 residues (CmNIP6;1), with an average of 276 aa. Finally, the SIP sub-family (with only two members) had a mean length of 275 aa. Motifs structure of each AQP of the same subfamily showed similar motifs structures (PIPs, TIPs and NIPs), NPA motif, and a large motif (YRALIAEFIATLLFLFVGVLTVIGYSKQTDTASLGGIG) conserved in all the sequences. However, SIPs and XIP1.1 showed different motif distribution, with some similarities between themselves. (Supplementary Figure S3).

Table 2 also displays the Pi and Mw data. The Pi varied among the families but was more or less constant among the sub-family members, with some exceptions. The PIPs sub-family had an average Pi of 8.58. In contrast, the TIPs group showed a mean Pi of 6.08, the lowest of all the sub-families, but CmTIP5.1 stood out with a Pi of 8.31, quite high for this group. The median Pi was 7.81 in the NIPs and, lastly, the SIPs displayed the highest Pi of all the sub-families, with an average of 9.63. The Mw did not vary much among the sub-families, unlike the Pi. The PIPs, NIPs and SIPs had mean values of 30.27 kDa, 29.09 kDa and 29.32 kDa, respectively; only the TIPs presented a notable difference, with an average of 26.39 kDa.

**Figure 3:**
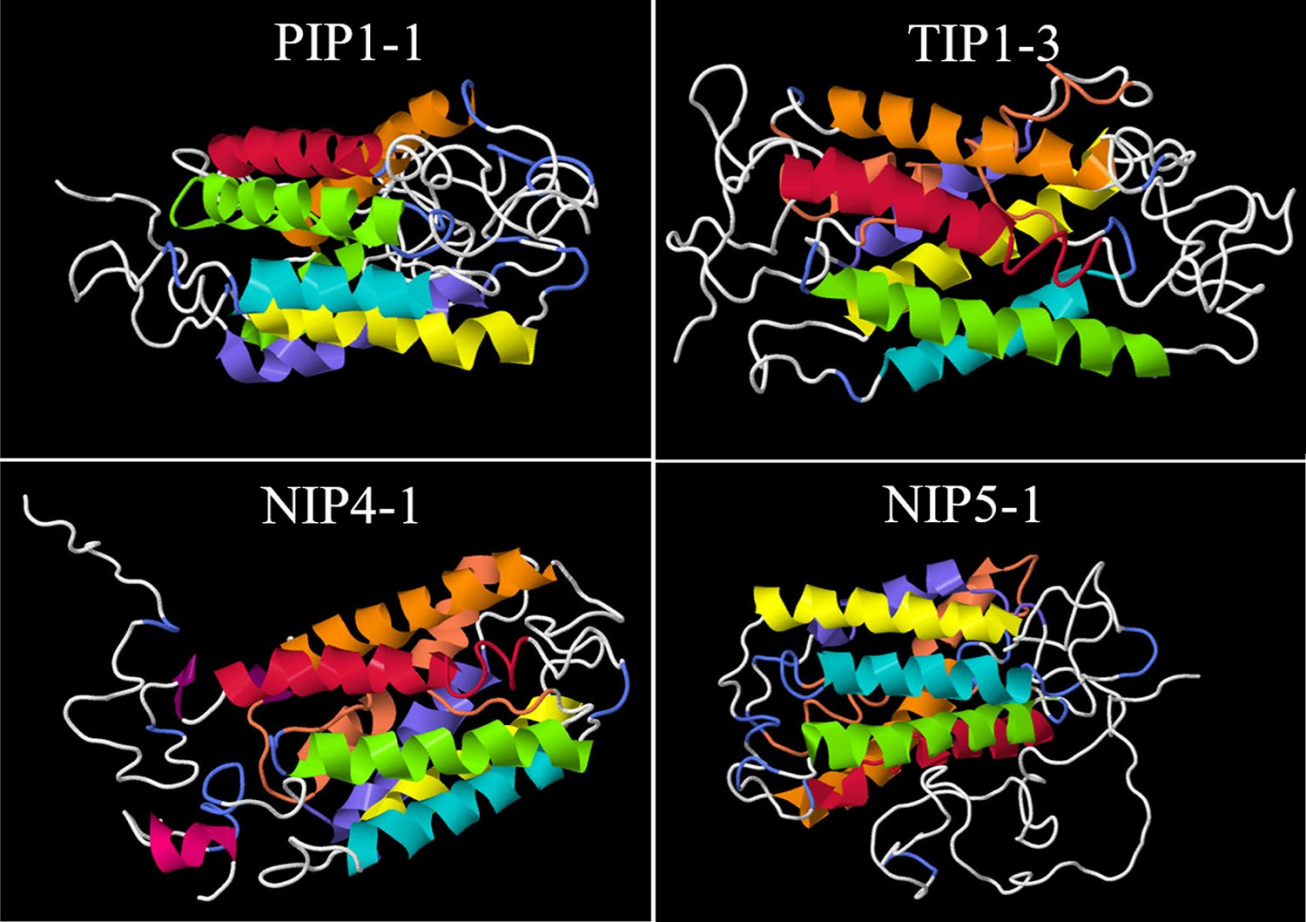
3D protein structure prediction for *C. melo* aquaporins: CmPIP1.1, CmTIP1.3, CmNIP4.1 and CmNIP5.1. Identification of the six transmembrane helices (H1–H6) and the additional two re-entrant helices are highlighted with different colours (H1 fuchsia, H2 green, H3 orange, H4 yellow, H5 sky blue, H6 dark blue, Re-entrant helices salmon). Protein structures were performed using Jmol software.

One of the principal features that define not only the aquaporins but also Integral Membrane Proteins (IMPs) is the presence of transmembrane helices. In the case of aquaporins, they must number at least six. We present, in Table 2, a prediction of the possible number of transmembrane motifs of each protein. For four of them, only five transmembrane helices were predicted and in order to confirm these results, we analysed the 3D structures of these proteins (Fig. 3); it can be seen that these four proteins displayed six transmembrane helices.

Prediction of the subcellular localisation, based on bioinformatics tools (the Plant-mPLoc and Wolf-PSORT programs), predicted all the PIP, SIP and XIP aquaporins situated on the plasma membrane. Most of the TIPs were predicted in the tonoplast, with certain exceptions: The Wolf-PSORT program predicts CmTIP1;1 in the plasma membrane, CmTIP3;1 in the mitochondrial membrane and CmTIP5.1 in the chloroplast membrane. As for the NIPs, almost all were predicted to have a plasma membrane localisation—except CmNIP2;1, CmNIP4;1, CmNIP5;2 and CmNIP6;1, which Wolf-PSORT placed in the tonoplast.

### Study of functional residues in aquaporins and possible solute transport

The functions of aquaporins are highly delimited by a few aa residues, mostly the NPA motifs, ar/R filter and FPs. These functional residues are listed in Table 3. The AQP phylogenetic framework can be used to predict the putative function of individual AQPs on the basis of orthologous genes from *A. thaliana* and other species^11^. Thus, the identification of conserved motifs in each subfamily and in each cluster of orthologous genes offers a framework for studying their possible functional implication^57^. Based on this principle, a comparative analysis of the main important residues of orthologous genes in *C. melo* and *A. thaliana* and their homologues in other species such as rice and maize was performed (Supplementary Table S1), as the transport of different solutes by their aquaporins has been studied previously, both functionally and experimentally. In addition to this comparison of homologues, mutational studies, pore structure analyses and experimental studies were considered, to propose the transport possibilities of each *C. melo* aquaporin, specified in Table 3.

**Table 3:** Identification of important residues and transport prediction for *C. melo* (Cm) aquaporins.

Gene name	NPA motif	ar/R selectivity filter				Froger’s positions					Transport prediction
	LB/LE	H2	H5	LE1	LE2	P1	P2	P3	P4	P5	
*CmPIP1;1*	NPA/NPA	F	H	T	R	Q	S	A	F	W	CO_2_^abc^, H_2_O_2_^ab^, B*^1^
*CmPIP1;2*	NPA/NPA	F	H	T	R	E	S	A	F	W	CO_2_^b^*^2^, H_2_O_2_^b^, Urea *^1^
*CmPIP2;1*	NPA/NPA	F	H	T	R	Q	S	A	F	W	CO_2_^ab^, H_2_O_2_^ac^
*CmPIP2;2*	NPA/NPA	F	H	T	R	Q	S	A	F	W	CO_2_^ab^, H_2_O_2_^ac^
*CmPIP2;3*	NPA/NPA	F	H	T	R	Q	S	A	F	W	CO_2_^ab^, H_2_O_2_^ac^
*CmPIP2;4*	NPA/NPA	F	H	T	R	Q	S	A	F	W	CO_2_^ab^, H_2_O_2_^ac^
*CmPIP2;5*	NPA/NPA	F	H	T	R	Q	S	A	F	W	CO_2_^ab^, H_2_O_2_^a^
*CmPIP2;6*	NPA/NPA	F	H	T	R	Q	S	A	F	W	CO_2_^ab^, H_2_O_2_^a^
*CmPIP2;7*	NPA/NPA	F	H	T	R	Q	S	A	F	W	CO_2_^ab^, H_2_O_2_^a^
*CmPIP2;8*	NPA/NPA	F	H	T	R	Q	S	A	F	W	CO_2_^ab^, H_2_O_2_^a^
*CmPIP2;9*	NPA/NPA	F	N	A	R	K	S	A	F	W	–
*CmPIP2;10*	NPA/NPA	F	H	T	R	M	S	A	F	W	CO_2_^abc^, H_2_O_2_*^2^, As^c^*^3^
*CmTIP1;1*	NPA/NPA	H	I	A	V	T	A	S	Y	W	H_2_O_2_*^2^, NH_3_^j^*^2^, Urea^b^*^2^, B*^1^
*CmTIP1;2*	NPA/NPA	H	I	A	V	I	A	A	Y	W	H_2_O_2_*^2^, NH_3_^j^*^2^, Urea^b^*^2^, B*^1^
*CmTIP1;3*	NPA/NPA	H	I	A	V	T	T	A	Y	W	H_2_O_2_*^2^, NH_3_^j^*^2^, Urea^b^*^2^, B*^1^ Gly*^3^
*CmTIP2;1*	NPA/NPA	H	I	G	R	T	S	A	Y	W	H_2_O_2_^ac^, NH_3_^abcghj^, Urea^abcdg^
*CmTIP2;2*	NPA/NPA	H	I	G	R	T	S	A	Y	W	H_2_O_2_^ac^, NH_3_^abcghj^, Urea^abcdg^
*CmTIP3;1*	NPA/NPA	H	I	A	R	T	A	S	Y	W	NH_3_^gj^, Urea^bd^
*CmTIP4;1*	NPA/NPA	H	I	A	R	T	S	A	Y	W	H_2_O_2_^a^, NH_3_^ahj^, Urea^abd^*^2^
*CmTIP5;1*	NPA/NPA	N	V	G	C	I	A	A	Y	W	Urea^d^
*CmNIP1;1*	NPA/NPA	W	V	A	R	F	S	A	Y	I	H_2_O_2_^a^*^1^*^2^, As^c^*^2^*^3^, Sb*^2^, B*^1^, Gly^ce^*^1^*^2^
*CmNIP2;1*	NPA/NPV	G	S	G	R	L	T	A	Y	F	Si^abik^*^1^*^3^, H_2_O_2_*^1^, As^ac^*^1^*^3^, Sb^ak^*^1^*^3^, Urea^abc^*^3^, B^bck^*^1^*^3^, Gly^c^*^1^
*CmNIP2;2*	NPA/NPA	C	S	G	R	L	S	A	Y	M	Si^k^, As^k^, B^k^
*CmNIP4;1*	NPA/NPA	W	V	A	R	L	T	A	Y	I	Gly^e^
*CmNIP5;1*	NPS/NPV	A	I	G	R	F	T	A	Y	L	As^ack^*^2^, Sb^a^*^2^, B^ack^*^2^, Urea^f^, Gly^cf^
*CmNIP5;2*	NPS/NPV	S	I	G	R	F	T	A	Y	L	–
*CmNIP6;1*	NPA/NPV	T	V	A	R	F	T	A	Y	L	Sb^a^
*CmNIP7;1*	NPA/NPA	A	V	A	R	F	S	A	Y	I	Urea^f^, Gly^f^
*CmSIP1;1*	NPT/NPA	F	I	P	N	M	A	A	Y	W	–
*CmSIP2;1*	NPL/NPA	I	H	G	S	F	V	A	Y	W	–
*CmXIP1;1*	SPI/SPA	I	I	V	R	M	C	A	F	W	–

Columns: gene name; NPA motif (LB and LE positions); ar/R selectivity filter (H2, H5, LE1, LE2), Froger’s positions (P1–P5) and transport prediction.

Shortening codes: LB, loop B; LE, loop E; H, hélix; Si, silicon; B, boron; As, arsenic; Sb, antimonite; Gly, glycerol. Aminoacide residues named with letters according the international code.

^a^Transport prediction according to Azad et al.^12^.

^b^Transport prediction according to Hove and Bhave^10^.

^c^Transport prediction according to Perez Di Giorgio et al.^11^.

^d^Transport prediction according to Dynowski et al.^28^.

^e^Transport prediction according to Wallace et al.^29^.

^f^Transport prediction according to Wallace and Roberts^27^.

^g^Transport prediction according to Azad et al. (2011).

^h^Transport prediction according to Kirscht et al.^74^.

^i^Transport prediction according to Deshmukh et al.^85^.

^j^Transport prediction according to Jahn et al.^30^.

^k^Transport prediction according to Mitani-Ueno et al.^26^.

*^1^ prediction based on *Zea mays *L. aquaporins orthologues and homologues.

*^2^ prediction based on *Arabidopsis thaliana *L. aquaporins orthologues and homologues.

*^3^ prediction based on *Oryza sativa *L. aquaporins orthologues and homologues.

### Expression analysis

After primer verification, the expression of 31 aquaporins of *C. melo* was analysed in root and leaf tissue by RT-qPCR. All aquaporins were detected in both tissues, except *PIP2;9*, which was only detected in roots, and *XIP1;1*, that could not be detected in any sample.

**Figure 4:**
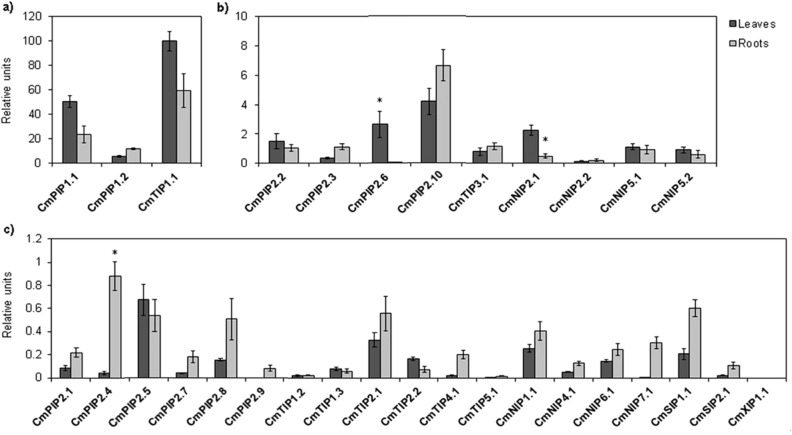
Expression levels of aquaporins genes. All the analyses were performed using qPCR, for leaves and roots. Statistical analysis was performed using SPSS 25.0.0.1. The values are the means ± s.e.m. of 3 to 6 biological replicates. Columns with * differ significantly according to Tukey’s test (*p* = 0.05). Each group is formed by aquaporins genes with similar expression levels represented as relative units (ru) (**a**) Group 1 (10–100 ru): *PIP1;1, PIP1;2* and *TIP1;1* (**b**) Group 2 (1–10 ru): *PIP2;2, PIP2;3, PIP2;6, PIP2;10, TIP3;1, NIP2;1, NIP2;2, NIP5;1* and *NIP5;2* (**c**) Group 3 (0–1 ru): *PIP2;1, PIP2;4, PIP2;5, PIP2;7, PIP2;8, PIP2;9, TIP1;2, TIP1;3, TIP2;1, TIP2;2, TIP4;1, TIP5;1 NIP1;1, NIP4;1, NIP6;1, NIP7;1, SIP1;1*, *SIP1;2* and *XIP1;1.* This figure was made using Microsoft Office 2016 package.

Figure 4 shows the aquaporins grouped according to their levels of expression (*see Material and Methods*). Group 1 contains the aquaporins that showed higher expression (10–100 ru) in both tissues: *TIP1;1, PIP1;2* and *PIP1;1* (Fig. 4a). Group 2 contains those with medium levels of expression (1–10 ru): *PIP2;2, PIP2;3, PIP2;6, PIP2;10, TIP3;1, NIP2;1, NIP2;2, NIP5;1 and NIP5;2* (Fig. 4b). Finally, group 3 contains the aquaporins *PIP2;1, PIP2;4, PIP2;5, PIP2;7, PIP2;8, PIP2;9, TIP1;2, TIP1;3, TIP2;1, TIP2;2, TIP4;1, TIP5;1 NIP1;1, NIP4;1, NIP6;1, NIP7;1, SIP1;1, SIP1;2 and XIP1;1*, which showed low levels of expression (0–1 ru) (Fig. 4c).

The aquaporin that showed the highest gene expression in the analysed tissues was *TIP1;1*. Among the PIP subgroups, the PIP1s clearly showed higher expression levels, *PIP1;1* being stronger in the leaves and both *PIP1;1* and *PIP1;2* in the roots, while of the PIP2s the most-highly expressed was *PIP2;10*, in both roots and leaves, this latter tissue also showing high expression of *PIP2;6*. Nor should the presence of *PIP2;2* and *PIP2;3*, in both tissues, and of *PIP2;4*, in roots, be neglected. Within the TIPs subfamily, *TIP1;1* exhibited the highest expression in both roots and (especially) leaves, followed by *TIP3;1*, in both tissues. Attending to the NIPs groups, the most expressed were *NIP2;1*, *NIP2;2*, *NIP5;1* and *NIP5;2*; the expression of *NIP2;1* was more important in leaves while both *NIPs5* and *NIP2;2* were expressed in roots and leaves at similar levels. Regarding the rest of the aquaporins, it should be noted that most of them appeared to a greater extent in the roots, being either lower, or practically nil, their presence in leaves, with no significant differences between them. As exceptions, *PIP2;5*, *TIP2;2* and *TIP1;3* had practically the same level of expression in both tissues, although slightly higher in leaves.

### Comparison with RNA-seq expression

**Figure 5:**
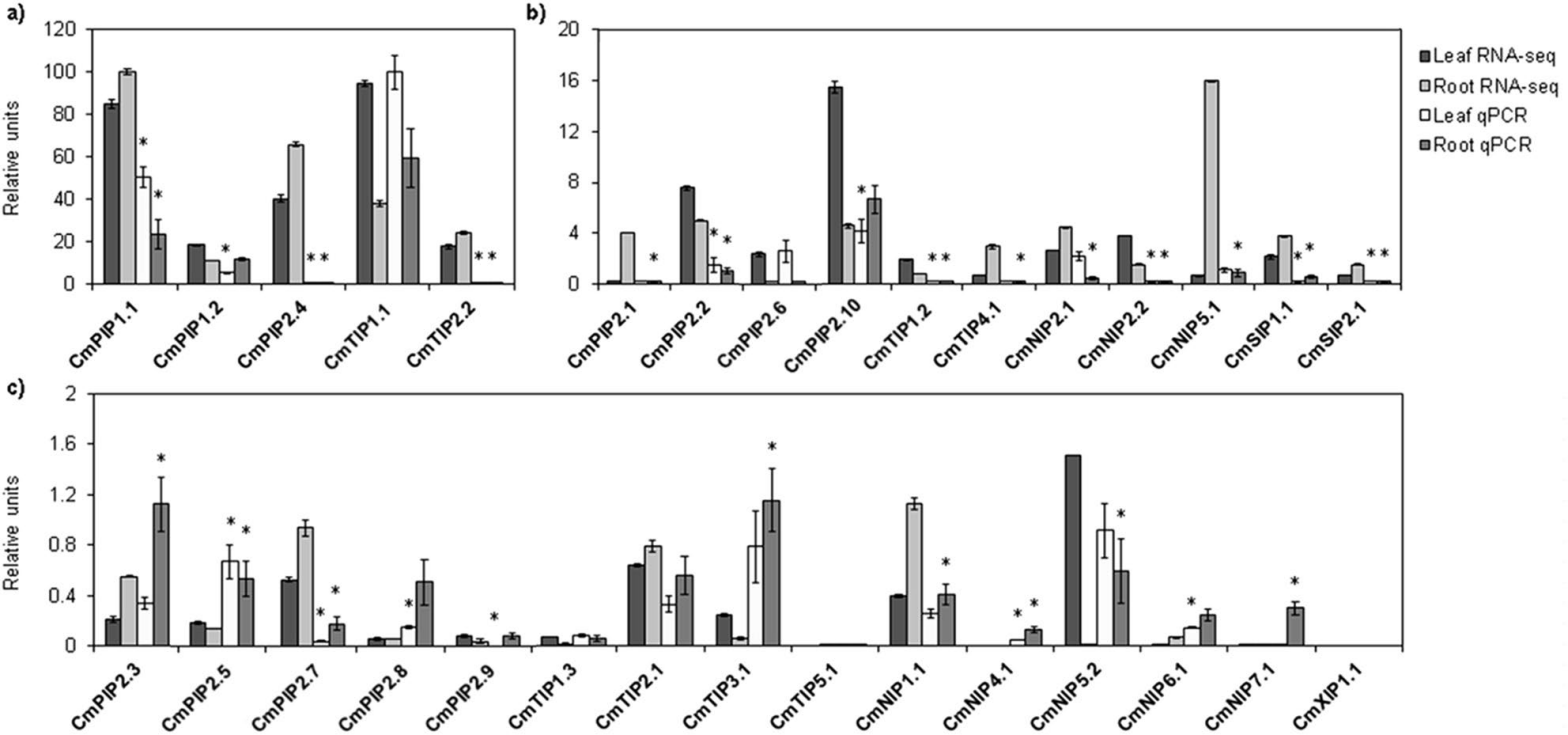
Comparison of the expression levels of aquaporins genes determined by qPCR with RNA-seq analysis in leaves and roots. Statistical analysis was performed using SPSS 25.0.0.1. Each bar represents the mean of 2 biological replicates for the RNA-seq data, and of 3 to 6 biological replicates for RT-qPCR. Columns with * differ significantly according to Tukey’s test (*p* = 0.05). Each group is formed by aquaporins genes with similar expression levels represented as relative units (ru). (**a**) Group 1 (20–120 ru): *PIP1;1, PIP1;2 PIP2;4, TIP1;1* and *TIP2;2.* (**b**) Group 2 (2–20 ru): *PIP2;1, PIP2;2, PIP2;6, PIP2;10, TIP1;2, TIP4;1, NIP2;1, NIP2;2, NIP5;1, SIP1;1* and *SIP1;2*. (**c**) Group 3 (0–2 ru): *PIP2;3, PIP2;5, PIP2;7, PIP2;8, PIP2;9, TIP1;3, TIP2;1, TIP3;1, TIP5;1*, *NIP1;1, NIP4;1, NIP5;2, NIP6;1, NIP7;1* and *XIP1;1.* This figure was made using Microsoft Office 2016 package.

After our expression analysis, we decided to compare the expression of 31 aquaporins of *C. melo* var. Grand Riado with the RNA-seq of another variety, in this case Cantalupo (cantaloupe)^56^. The comparative analysis of the two techniques highlighted three different behaviour patterns (Fig. 5). Some aquaporins had very similar levels in both root and leaf tissues, such as *PIP2;6, TIP1;1, TIP2;1, TIP1;3, TIP5;1* and *XIP1;1.* Other aquaporins had similar levels in one tissue: like *PIP1;2, PIP2;9 and PIP2;10* in roots, and *PIP2;1, PIP2;3, TIP3;1, TIP4;1, NIP1;1, NIP2;1, NIP5;1, NIP5;2* and *NIP7;1* in leaves. By contrast, we obtained completely different patterns for other aquaporins: namely, *PIP1;1, PIP2;2, PIP2;4, PIP2;7, TIP1;2, TIP2;2, NIP2;2, SIP1;1* and *SIP2;1*, which all had lower expression in our analysis than in the RNA-seq analysis, and *PIP2;5, PIP2;8, NIP4;1* and *NIP6;1*, which all had higher expression in our analysis.

Based on the RNA-seq analysis, *TIP1;1* and *PIP1;1* were the most expressed aquaporin isoforms in melon plants (Fig. 5a). Both PIP1s were among the aquaporins with more presence in both tissues (roots and leaves). Of the PIP2 isoforms, *PIP2;4* was highly expressed, especially in root tissues, closely followed by *PIP2;10*, with a strong presence in leaves. Other prominent isoforms within this subgroup were *PIP2;1*, *PIP2;2* and *PIP2;6* (Fig. 5b). Within the TIPs subfamily, *TIP2;2* seemed to be important in both tissues (Fig. 5a). Attending to the NIPs subfamily, *NIP5.1*, *NIP2.1* and *NIP2.2* were the most expressed, being very striking the high level of *NIP5.1* in roots (Fig. 5a). Comparing the NIP2s, *NIP2.1* was expressed more in roots while *NIP2.2* was higher in leaf tissue (Fig. 5b). Of the SIPs, *SIP1.1* was the most expressed, its presence being more important in roots (Fig. 5b).

## Discussion

The availability of the whole genome sequence of melon would facilitate genome-wide analysis to identify the complete set of aquaporins. Also, some analyses of the expression of diverse aquaporins with RNA-seq techniques have been performed; thanks to these it is possible to find in the various databases the nucleotide sequences of a large number of melon aquaporins genes, although many of them come from diverse origins and have not been ordered, compared and classified. In this way, the number of aquaporins genes identified in melon (31) was comparable with the number found in other plant species, such as rice (33)^49^, *A. thaliana* (35)^2^ and watermelon (35)^20^. It seems that some aquaporin genes physically cluster very close to each other in certain regions, suggesting the occurrence of more than one gene tandem duplication event in the evolutionary history of melon, as was previously studied in tobacco plants^58^. There is an accumulation of the PIP2 genes in chromosome 5. In the same way, the co-location of *CmNIP2;1* and *CmNIP2;2* in chromosome 4 and of *CmNIP5;1* and *CmNIP5;2* in chromosome 9 could allude also to genomic duplication events. Despite this, further investigations are needed to clarify the evolutionary events that have occurred across the aquaporin family in *C. melo*.

The protein features, such as the mean Mw, of each subfamily also showed similarities to those of *A. thaliana*^59^. However, a notable difference is shown in the SIPs subfamily, the Mw being greater in melon than in *A. thaliana*; this could be due to the larger sequence found in *CmSIP2;1*, compared with *AtSIP2;1*. Furthermore, when the protein sequence of CmSIP2;1 was compared with those of homologous SIP2;1 proteins in other species (watermelon, arabidopsis and cucumber)^2,20,47^, all the sequences aligned from the second methionine residue of CmSIP2;1. This suggests that this site is the starting point of translation, resulting in a protein of 238 aa and 25.99 kDa, thus equating the results to those obtained in arabidopsis for this subgroup. However, this hypothesis needs to be studied in order to clarify all the protein features previously predicted. In addition, TIPs were not found to be smaller than PIPs and NIPs, but most of them were more acidic (with lower Pi) than PIPs, NIPs, SIPs and XIP. The Pi could reflect a functional constraint imposed on MIPs^60^. Indeed, sequence analysis in arabidopsis revealed that the cause of this difference in Pi lies in the C-terminal regions, which are more basic in PIPs and NIPs than in TIPs. Therefore, it is possible that phosphorylation sites or sorting signals in the C-terminal regions form part of the hypothesised functional constraint on the sequences^60^.

The aquaporins monomers found a highly conserved structure, with six transmembrane helices in all CmAQPs (Table 1, Fig. 3). These strongly conserved regions has been reported to be likely constrained to maintain the structural integrity of the aquaporin monomer and the conservation of critical residues which was essential for tetramer formation^61^. In this way the subcellular location of aquaporins, PIPs, NIPs and XIPs are usually located in the plasma membrane, while TIPs and SIPs are normally localised to the tonoplast and endoplasmic reticulum (ER), respectively^62,63^. However, our results showed that the PIPs, NIPs, SIPs and XIP were located principally in the plasma membrane (Table 2). This was expected, with the exception of the SIPs—that were not located in the ER by any of the programs used, although this is their most probable location. Nevertheless, a few of NIP proteins were located not only in the plasma membrane but also in the tonoplast and the TIPs showed a diverse range of possible subcellular locations depending on the chosen prediction software (chloroplast, mitochondria, plasma membrane and cytoplasm). Significantly, CmTIP5;1 was the only aquaporin assigned to chloroplasts, while CmTIP3;1 was localized in mitochondria (Table 2). As cell compartmentalization has represented the main driving force in the diversification of aquaporins in plants, no location should be excluded without specific analysis and more studies must be made to confirm these preliminary data. Nevertheless, NIPs isoforms have not yet been found in the tonoplast, so this does not appear to be their most likely location. A role for AtTIP5;1 as a nitrogen transporter in mitochondria has been proposed^64^ and different isoforms of PIPs and TIPs have been detected in chloroplasts^65,66^; a role in the transport of CO_2_, water and H_2_O_2_ in chloroplast membranes could be beneficial to the plant dynamics^62^. So, the predicted localization of CmTIP3;1 in mitochondria and of CmTIP5;1 in chloroplasts cannot be rejected, even if this location for CmTIP5;1 does not seem to coincide with the gene expression data (Fig. 4c), which place it mostly in roots.

### Analysis of possible functions in solutes transport (Table 3)

The function of PIPs in water transport is very specialised and their structure is highly preserved^67^. All PIPs have the same NPA motifs and ar/R selectivity filters residues (F, H, T and R in H2, H5, LE1 and LE2, respectively) (Table 3), directly related to their main role in water transport^7^, while phylogenetic and functional studies suggest that they are equally capable of transporting CO_2_^10,12^. The FPs are also very similar in most PIP aquaporins from both subgroups (PIP1s and PIP2s). The S-A-F-W residues are well conserved in all cases in positions P2 to P5 respectively. In nine of the 12 PIPs, P1 was a Q (Gln) residue; the exceptions were CmPIP1;2 with E (Glu), CmPIP2;9 with K (Lys) and CmPIP2;10 with M (Met) (Table 3). The comparison of melon FPs with homologues in other species predicted the possible transport of H_2_O_2_ by almost all melon PIPs^12^. The only exception was the unusual CmPIP2;9, whose aa residues in the ar/R filter (F-N-A-R) and in the P1-FPs (K) differed from those of the PIP2s characterised to date and whose particular sequence has not been previously described. The sequence homology analysis (Supplementary Table S1) of PIPs that have been shown to transport B, urea and As resulted in the prediction of matching transport by orthologues in *C. melo*^11^. According to this, CmPIP1s could be able to transport boric acid and/or urea, like their orthologues ZmPIP1;1 and ZmPIP1;5^31,32^, while CmPIP2;10 could transport As, as OsPIP2;6 does^45^ (Supplementary Figure S2).

Based on the ar/R filter^68^, the CmTIPs were classified in four groups attending to their homology. Group I is formed by CmTIP1;1, CmTIP1;2 and CmTIP1;3, Group IIa is formed by CmTIP2;1 and CmTIP2;2, Group IIb is constituted by CmTIP3;1 and CmTIP4;1 and Group III has only one member, CmTIP5;1. In general, TIPs seem to have developed the capacity to transport nitrogenous compounds^69^. The great importance of the H2 and H5 positions (H–I) with a non-polar LE1 (A/G) in the groups I, IIa and IIb has been related with NH_3_ transport^30^. Many TIPs have been characterised as urea transporters^32,33,46,70^. In the TIPs group I, the substitution of the typical R by V in the LE2 position has been proved to be involved in the transport of not only NH_3_ and but also H_2_O_2_, in mutagenic studies^30,71,72^. Regarding the FPs, the CmTIP1s presented slight differences, and a direct homology with residues of the other plants studied was not found (Supplementary Table S1). However, the phylogenetic analysis clearly related the CmTIP1s with the TIP1s of *A. thaliana* (Fig. 1). All the AtTIP1s were predicted to transport urea and H_2_O_2_^10–12^ and this has been proven experimentally in heterologous systems^33,36,70^, while NH_3_ transport has only been tested in mutagenic studies^72^. All this suggests that *C. melo* TIP1s are able to transport these solutes. The clade including CmTIP1;3, AtTIP1;3, ZmTIP1;2 and OsTIP1;2 diverged earlier, followed by the separation of CmTIP1;2 that presents a greater evolutionary divergence from AtTIP1s but is closer to OsTIP1;1 and ZmTIP1:1 (Supplementary Figure S2). Interestingly, it has been shown that OsTIP1;2 can transport glycerol^73^ and that ZmTIP1;1 and ZmTIP1;2 can transport boric acid, in addition to NH_3_, urea and H_2_O_2_^46^. So, CmTIPs1 might be able to transport B and CmTIP1;3 might be able to transport Gly also (Table 3). Regarding Group IIa (TIP2s), their ar/R region has been directly related to the transport of NH_3_ and urea^28,33,72,74^. A specific amino acid, histidine (H), in loop C seems to be key for the de-protonation of NH_4_^+^, which would allow the transport of NH_3_ independent of pH^74^. This residue is present in CmTIP2s and CmTIP4.1, but not in the other TIPs subgroups, which lack the ability to extract a proton from an NH_4_^+^ ion; thus, the passage of ammonia relies on the pH-dependent concentration of uncharged NH_3_ in the medium^71^. The transport predictions for FPs indicate that they can transport not only NH_3_ and urea but also H_2_O_2_^10–12^; their relationship with AtTIP2s (Fig. 1) also suggests this capability, while the ability of orthologues to transport H_2_O_2_ has only been tested in assays with mutants^75^. Phylogenetic analysis of the TIPs group IIb showed that CmTIP3;1 is closely related to AtTIP3s (Fig. 1), although there is a substitution in FPs P3 (A is replaced by S) (Table 3). The ar/R analysis supports the NH_3_ and urea transport capacity^28,30,72^. As stated previously, CmTIP4;1 and CmTIP2s share all FPs, the His in loop C^74^ and the same ar/R region (with the only exception of G instead of A in LE1) (Table 3), pointing to selective NH_3_ transport. Urea transport by CmTIP4;1 is supported by mutant studies^28^ and has been shown to be transported by the orthologous AtTIP4.1^33^. The prediction based on the phylogenetic framework, and including FPs, also indicated the ability to transport H_2_O_2_^12^. CmTIP5;1 is the only aquaporin in group III and its orthologue AtTIP5;1 has been shown to transport urea^70^. Their FPs differ only in the P1 position (Table 3), which in *C. melo* is a residue of I instead of V (both non-polar); therefore, the transport of urea by CmTIP5;1 seems to be possible and is supported by mutagenic analysis of the ar/R region^28^.

The NIPs form a monophyletic group^76^ and overwhelming evidence supports a main role for NIPs in metalloids transport^77^ beside glycerol—including B, Si, As and Sb [reviewed in Pommerrenig et al. (2015)]. Interestingly, all these compounds, in their uncharged states, have a geometry that is substantially similar to a conformation of glycerol in a retracted state; this would readily allow adaptation of the pore of the original aquaglyceroporin NIPs to the transport of these compounds^78^. Eight NIPs have been found in *C. melo* and have been classified into the three subgroups of NIPs based on their ar/R filter^79^. CmNIP1;1 and CmNIP4;1 belong to Group I. CmNIP5;1, CmNIP5;2, CmNIP6;1 and CmNIP7;1 belong to group II, although some of their compositions vary from the typical ar/R residues (discussed below), and CmNIP2;1 and CmNIP2;2 belong to Group III. The typical W-V-A-R in H2, H5, LE1 and LE2, respectively, are the characteristic ar/R residues in Group I and have been directly related to water and glycerol transport^29^. Both CmNIP1;1 and CmNIP4;1 have these characteristic motifs (Table 3). The phylogenetic tree places both in the same clade along with AtNIP1;1, AtNIP1;2, AtNIP2;1 and AtNIP3;1 (all in a branch with CmNIP1;1), AtNIP4;1 and AtNIP4;2 (both in the same branch as CmNIP4.1) (Fig. 1). Experimental studies showed that AtNIP1s were able to transport As, Sb, H_2_O_2_ and Gly^37,42,75,80^. CmNIP1;1 appears to have separated from AtNIP1s earlier, in an evolutionary branch that it shares with maize and rice NIP1s (Supplementary Figure S2). OsNIP1;1 can transport As^38^ while ZmNIP1;1 has been shown, in heterologous systems, to transport H_2_O_2_ and glycerol; unexpectedly, it is also able to transport boric acid efficiently^46^. Thus, CmNIP1;1 might transport As, Sb, H_2_O_2_ and Gly, while B cannot be ruled out. Interestingly, while CmNIP4;1 belongs to group I and has the same ar/R motifs, its FPs are more similar to those of NIPs group II. This increases the possibility that it transports other interesting solutes, such as those that can be transported by NIP2s (discussed below). Despite this, the homology-based prediction did not allow a specific transport role to be assigned to CmNIP4;1, beyond glycerol transport compatibility^29^, due to its clear differences in FPs from its orthologues (Supplementary Figure S2 & Supplementary Table S1). Strong evidence supports the role of the NIPs II group in boric acid transport^34,41,81^. Among the *C. melo* NIPs belonging to this group, the most important variation is in H2; in CmNIP5;2 it is an S residue and in CmNIP6;1 a T residue (Table 3). However, both CmNIP5;2 and CmNIP6;1 conserve FPs identical to those of CmNIP5;1 and homologous to those of other aquaporins of group II found in arabidopsis, rice and maize (Supplementary Table S1), all of them belonging to the same clade in the phylogenetic tree (Supplementary Figure S2). CmNIP5;1 and CmNIP5;2 are very close to AtNIP5;1, which has been proved to transport As, Sb and B in experimental assays^26,34,82^. CmNIP6;1 is related directly to AtNIP6;1, the latter being able to transport not only As, Sb and B but also urea and Gly^27,41,82^ (Fig. 1). The prediction based on the phylogenetic framework, and including FPs, assigned to CmNIP5;1 the ability to transport As, Sb and B, as AtNIP5;1 does. This As and B transport is supported by mutagenic analysis^26^ and its ability to transport Gly and/or urea must also be considered^27^. The variation in H2 of CmNIP5;2 (Table 3) prevented the prediction of any transport ability, although it should not be ruled out that it fulfils functions similar to those of CmNIP5;1. The same applies to CmNIP6;1, for which the prediction points only to Sb transport, although this is most likely not its only function. Regarding CmNIP7;1, its phylogenetic divergence does not separate it from group II of the NIPs and its proximity to AtNIP7;1 suggests that it can perform similar functions (Fig. 1). AtNIP7;1 can transport As, Sb, B, urea and Gly^73,82^. The possible transport of Gly and/or urea is also supported by studies with point mutations^27^ and by the presence of a specific Y64 residue (Y81 in AtNIP7;1) which has been directly related to gating and regulation of urea and Gly transport^83^. The aquaporins CmNIP2;1 and CmNIP2;2 are included in the NIP III group^79^. They possess a unique ar/R selectivity filter due to the small size of the aa in the H2 and H5 positions, giving the largest pore diameter described in aquaporins, that may allow the passage of very large solutes such as Si (4.38 Å)^84^. Indeed, the transport of Si has been specifically associated with the motifs that are present in CmNIP2;1^85^. Si transporters are absent from the Brassicaceae and, therefore, NIP III aquaporins have not been found in *A. thaliana*, but they are present in all silicon-accumulator plants. Phylogenetic analysis assigned CmNIP2;1 and CmNIP2;2 to the same clade as the *Z. mays* and *O. sativa* NIP2s (Supplementary Figure S2), strongly suggesting that all members of the *C. melo* NIPs III group are Si transporters. In addition to silicon, members of the NIPs group III from *Z. mays* and *O. sativa* have been proved experimentally to transport As, Sb, B and urea^39,46,79,86–88^; also, ZmNIP2:1 was able to transport small amounts of H_2_O_2_ and Gly in heterologous expression experiments^46^. Thus, according to the analysis of orthologues, the transport of most of these solutes could occur via *C. melo* NIP2s and the prediction based on homologies also points to such transport by CmNIP2;1^10–12^. The case of CmNIP2;2 is peculiar since it clearly belongs to the same protein family but has a variation in the H2 position of the ar/R selectivity filter (C instead of G), and also its FPs are slightly different (S in P2, instead of T) from those of the NIPs III group (Table 3); hence, a prediction could not be performed based on homologous genes. The substitution of G in H2 by another non-polar small residue (A) did not affect the ability of OsNIP2;1 to transport Si, As or B^26^, so it is reasonable to propose that CmNIP2;2 can also transport some of these solutes.

Very little is known about the function of SIPs *in planta*. They are localised in the ER and facilitate water transport^89^ but no data on transport of non-aqueous substrates are available. Despite this, homologues- and structure-based analyses predict that the SIP1s of *A. thaliana*, *Z. mays* and *O. sativa* are capable of transporting urea, while the SIP2s could transport H_2_O_2_ and As^12^. Phylogenetic analysis showed that the SIPs of *C. melo* are clearly related to their orthologues in *A. thaliana* (Fig. 1), although the variation that both *CmSIP1;1* and *CmSIP2;1* show in H2 impairs the direct assignation of solutes transport to these proteins (Table 3).

The absence of XIPs in the Brassicaceae and monocots makes it impossible to compare the sequence found in *C. melo* with those of *A. thaliana*, *Z. mays* and *O. sativa*. For this reason, we compared the important residues with those of XIPs in other species^90^. *CmXIP1;1* belongs clearly to the XIP-A subfamily, which does not yet have known transport functions. The main particularity of *Cucumis* (both *sativus* and *melo*) XIPs is that they present an SPI/SPA motif rather than a NPA/NPA motif, while conserving other MIP-specific sequence features. It is usual to find variations in the first NPA motif of XIP1s—as they have been observed in other species, such as *Ricinus communis* L., *Lotus japonicus* L.*, Prunus persica* L. and *Glycine max* L*.*^90^—but it is quite odd to find a mutation in the second NPA motif. Also, in the first NPA motif, the A is substituted by I, which is also quite rare and has been observed only in *Cucumis* to date. It is still unknown what kind of functional effect this residue change might have on the protein.

### Constitutive expression of CmAQPs and possible implication in melon physiology

Our results clearly show that the main aquaporin expressed in *C. melo* was *CmTIP1;1*, by a wide margin with respect to the other isoforms (Fig. 4a). The water transport capacity has been maintained in TIPs, especially in the TIP1s, for which the magnitude stands out, being mostly responsible for the high permeability of the tonoplast^91^. *CmTIP1;1*, therefore, should be mainly responsible for the water transport between the cytoplasm and vacuole in melon plants. The other TIP1s in *C. melo* showed very slight expression (Fig. 4c). If this is due to a redundancy of functions because of the importance of *CmTIP1;1*, or to their specialisation in other tissues or environmental conditions, remains to be investigated. Just below *CmTIP1;1*, the PIP1s isoforms were the aquaporin genes most expressed in our studies, in both roots and leaves (Fig. 4a). Other studies showed patterns similar to ours. For example, in maize, in the central zone of the leaf, the aquaporin gene most expressed was shown to be *ZmPIP1;1*, with PIP1s contributing more than 70% of the expression of PIPs in leaves^92^. The characteristic structure of PIPs is also clearly compatible with CO_2_ and H_2_O_2_ transport besides that of water. So, certain PIP1 isoforms might specialise in such functions, depending on their location, and some PIP1s were shown to be directly related with CO_2_ transport in mesophyll cells and chloroplasts^66,92,93^. This allows us to propose a function of *CmPIP1s* in the transport of both water and CO_2_ in melon, which could explain their extremely high expression in leaves. The PIP2s isoforms, discounting their specific function as water transporters, have been related with ROS signalling through their regulation by and transport of H_2_O_2_^36,75,94^. Attending to the expression profile, *CmPIP2;6* seems to have also a fundamental role in leaves (Fig. 4b), as occurs in other species such as Arabidopsis, in which one PIP2 (*AtPIP2;6*) is predominant in leaves^95^, pointing to a possible implication in H_2_O_2_ or even CO_2_ transport besides that of water. Indeed, it has been shown that, despite the high homology among the different PIPs, some isoforms of plants possess the ability to transport water but not CO_2_ (*NtPIP2;1*) or vice versa (PIP1, *NtAQP1*), the proportion of each of them in the tetramer allowing the passage of water or CO_2_ to be regulated. This opens the door to the involvement of the fifth, central pore in the transport of gases and to possible regulation of the transport by competition between subunits in the formation of the tetramer^5^. How tetramers can be established preferentially with one concrete isoform, to regulate such transport, could be of interest in future studies to see if *CmPIP2;6* is preferentially selected in tetramers with PIP1s involved in CO_2_ conduction.

In addition, PIP1s also had high expression in roots along with *CmPIP2;10*, which is probably the main water transporter in *C. melo*, especially in roots, followed by *CmPIP2;2*, *CmPIP2;3* and *CmPIP2;4* (Fig. 4b,c). As we show in this study, and in general under optimal conditions, there is greater expression of aquaporins in the roots^51,96,97^, since these are the point of entry of water and nutrients into the plant, and the predominance of a specific PIP2 isoform also seems to be habitual. As an example, *ZmPIP2;5* is the main PIP gene expressed in the roots of maize and has been shown to be essential for water to cross the Casparian barrier in the exodermis^51^. The heterotetrameric conformation that includes PIP2 and PIP1 subunits has been shown to improve water transport, versus heterotetramers consisting exclusively of PIP2s^98,99^. This could be a valuable source of water transport regulation in the roots of melon plants as the interaction of the two isoforms is essential for PIP1s to access their active location in the plasma membrane^99^. Interestingly, the orthologues of *CmPIP1;1*, *CmPIP1;2* and *CmPIP2;10*, the most expressed PIP isoforms in melon roots, have been found to transport other solutes such as urea, B and As^31,32,45^. The occurrence of this transport through these isoforms in melon could be of great interest to understand their expression patterns. Apart from *CmTIP1;1*, within the TIPs, only *CmTIP3;1* seems to have a significant presence under optimal conditions, in both roots and leaves (Fig. 4b). Given that the prediction of the subcellular location (Table 2) places *CmTIP3;1* in the mitochondria and the well-known ability of TIPs to transport nitrogen compounds then, if this location is confirmed, they could also play a role in the transport of NH_3_ produced by photorespiration^63^. Attending to the CmNIPs, the expression of *CmNIP5;1* and *CmNIP5;2* was similar, being low but significant in both roots and leaves (Fig. 4b). Strong evidence supports the role of the NIPs II group in boric acid transport^34,41,81^ and this is probably the main function of the CmNIP5s. The members of the NIPs III group have been defined clearly as Si transporters^100^ and the CmNIP2s were the most expressed NIPs genes in melon plants, especially *CmNIP2;1*, whose expression was higher in leaf tissues, while *CmNIP2;2* was expressed mostly in roots (Fig. 4b). In rice it has been shown that *OsNIP2;1* is the main Si transporter involved in Si uptake, while *OsNIP2;2* is mostly implicated in the unloading of Si from xylem vessels in leaves^35,43^. Similarly, the stronger presence of *CmNIP2;1* in leaves suggests a Si-unloading function in leaves, while the two isoforms had the same expression in roots, implicating both in the uptake and translocation of Si in roots. However, although neither B transport by *CmNIP5;2* nor Si transport by *CmNIP2;2* could be directly predicted based on previous analyses (Table 3), these are probably their main functions. This idea is supported by the tight tandem that each of them forms with its respective paralog in chromosomes 9 and 5, respectively. These probably represent the result of gene duplication events that yielded differences in discrete functions or sub-localisations within the same tissue, or even between different tissues, as seems to have been the case in the NIP2s.

Finally, it is worth noting the expression of the aquaporin *CmSIP1;1* in roots, which was at the level of many PIPs and more than half of the isoforms analysed. Thus, although specific functions are not known, it seems to have some importance in the physiology of melon roots.

### Comparison of expression profiles in RNA-seq versus qPCR

Quantitative reverse transcription PCR (RT-qPCR) was the tool used most widely for the quantification of gene expression due to its sensitivity and precision^101^, being considered the most important medium-performance gene-expression analysis technology^102^, and it should be used to validate RNA sequencing, which is the most widely used tool today. Based on this, we decided to compare the expression of the 31 aquaporins genes of *C. melo* var. Grand Riado with the RNA-seq data available in the databases, which refer to another variety, in this case Cantaloupe (melon)^56^.

We found numerous similarities between our qPCR analyses and the RNA-seq data. Among them, the aquaporins with higher levels of expression showed better correlations for one or both tissues; this was the case for *PIP1;2, PIP2;1, PIP2;6, PIP2;10, TIP1;1, TIP4;1, NIP2;1* and *NIP5;1.* The differences in expression between the qPCRs and RNA-seq can be explained, since the sensitivity of the qPCRs decreases from cycle 34 onwards and most of the differences were found in aquaporins that appeared from this cycle onwards due to their low expression. Other mismatches between the two analyses can be explained by the use of different melon varieties. This could have led to changes in the expression pattern of some aquaporins—as seen in other plant varieties, where differences have been found, even between different cultivars^103^. Lastly, the growth conditions were different: Latrasse et al. (2017) used constant humidity (60%) and a temperature of 21–27 ºC in a glasshouse (for melon plants grown on rock compost) and also grew plants in the field (in soil), while we used hydroponic culture, at 60–80% humidity and a temperature of 20–25 ºC^56^.

Despite the discrepancies, a clear relationship is seen regarding the importance of certain isoforms in *C. melo*. Clearly, *CmTIP1;1* is revealed as the most important osmoregulator in the tonoplast under optimal conditions. The PIP1s are of greater importance, in both roots and leaves, *CmPIP1;1* being the predominant isoform. *CmPIP2;6* is very important in leaf tissues, while in the case of the variety Cantaloupe its function seems to be shared with *CmPIP2;10*, which had a strong presence in leaves. In roots, the Cantaloupe aquaporins isoforms that seem to have the major role in water uptake are *CmPIP2;4*, followed by *PIP2;10, PIP2;2* and, finally, *PIP2;1*. Of the NIPs, *NIP5;1* and the NIP2s are undoubtedly important in melon plants. In the variety Cantaloupe, the presence of *NIP5;1* is extremely striking in roots, being one of the most expressed isoforms, while in Grand Riado both NIP5 isoforms may share redundant functions. The genetic duplication of the NIP2s in a narrow chromosomal tandem and their matching transport abilities, together with their differentiation with respect to the expression in roots and leaves, suggest that they have acquired tissue-specific roles, these possibly being different in each of the two varieties. Finally, both analyses indicated that *SIP1;1* plays a constitutive and important, but still unknown, role. It seems that, in each variety, slight changes in the weight of different paralogs have developed and these changes could be related to the transport function and/or localisation. The abundant solute transport possibilities for each aquaporin isoform could have played an important role in the specialisation within each variety, depending on the needs of each of these two varieties throughout their evolution and development in different environments.

## Concluding remarks

In this work, the sequences of aquaporins genes have been assessed in melon. The comparative analysis with related plant species was very useful to predict the possible roles of some of the aquaporins in the transport of certain solutes. This analysis together with qPCR carried out in roots and leaves revealed the constitutive expression of almost all aquaporins genes in both organs. However, the role of each aquaporin must be elucidated in future work. Therefore, the exhaustive design of all the primers presented here is intended to serve as the basis for future research into all aquaporins in melon, regarding their roles in plant development and in the response to stress conditions. Finally, this work will serve as a basis for other researchers who wish to carry out a comprehensive analysis of the genome, explaining and clarifying a simple way to carry out this type of research.

## Supplementary Information

Supplementary Information.

## Data Availability

The seeds were kindly provided by SAKATA SEED IBERICA, S.L.U. and all data generated or analysed during this study are included in this published article and its supplementary information files. Original data from qPCR are available from the corresponding author on reasonable request.

## References

[1] Tyerman SD, Niemietz CM, Bramley H (2002). Plant aquaporins: multifunctional water and solute channels with expanding roles. Plant Cell Environ..

[2] Johanson U (2001). The complete set of genes encoding major intrinsic proteins in Arabidopsis provides a framework for a new nomenclature for major intrinsic proteins in plants. Plant Physiol..

[3] Luang S, Hrmova M (2017). Structural Basis of the Permeation Function of Plant Aquaporins. Plant Aquaporins: From Transport to Signaling.

[4] Hub JS, De Groot BL (2006). Does CO2 permeate through aquaporin-1?. Biophys. J..

[5] Otto B (2010). Aquaporin tetramer composition modifies the function of tobacco aquaporins. J. Biol. Chem..

[6] Murata K (2000). Structural determinants of water permeation through aquaporin-1. Nature.

[7] Sui H, Han BG, Lee JK, Walian P, Jap BK (2001). Structural basis of water-specific transport through the AQP1 water channel. Nature.

[8] Curticăpean M-C (2020). Plant Aquaporins. Acta Biol. Marisiensis.

[9] Froger A, Tallur B, Thomas D, Delamarche C (1998). Prediction of functional residues in water channels and related proteins. Protein Sci..

[10] Hove RM, Bhave M (2011). Plant aquaporins with non-aqua functions: deciphering the signature sequences. Plant Mol. Biol..

[11] Perez Di Giorgio J (2014). Prediction of aquaporin function by integrating evolutionary and functional analyses. J. Membr. Biol..

[12] Azad AK (2016). Genome-wide characterization of major intrinsic proteins in four grass plants and their non-Aqua transport selectivity profiles with comparative perspective. PLoS ONE.

[13] Bienert GP, Chaumont F (2011). Plant Aquaporins: Roles in Water Homeostasis, Nutrition, and Signaling Processes. Transporters and Pumps in Plant Signaling.

[14] Bárzana G, Carvajal M (2020). Genetic regulation of water and nutrient transport in water stress tolerance in roots. J. Biotechnol..

[15] Bárzana G (2020). Interrelations of nutrient and water transporters in plants under abiotic stress. Physiol. Plant..

[16] Fleshman MK (2011). Carotene and novel apocarotenoid concentrations in orange-fleshed cucumis melo melons: determinations of β-carotene bioaccessibility and bioavailability. J. Agric. Food Chem..

[17] Kolayli S (2010). Comparative study of chemical and biochemical properties of different melon cultivars: standard, hybrid, and grafted melons. J. Agric. Food Chem..

[18] Vouldoukis I (2004). Antioxidant and anti-inflammatory properties of a Cucumis melo LC. extract rich in superoxide dismutase activity. J. Ethnopharmacol..

[19] Preciado P (2018). Increasing doses of potassium increases yield and quality of muskmelon fruits under greenhouse. Horticultura.

[20] Zhou Y, Tao J, Ahammed GJ, Li J, Yang Y (2019). Genome-wide identification and expression analysis of aquaporin gene family related to abiotic stress in watermelon. Genome.

[21] Garcia-Mas J (2012). The genome of melon (*Cucumis melo* L.). Proc. Natl. Acad. Sci. USA.

[22] Krogh A, Larsson B, Von Heijne G, Sonnhammer ELL (2001). Predicting transmembrane protein topology with a hidden Markov model: application to complete genomes. J. Mol. Biol..

[23] Buchan DWA, Jones DT (2019). The PSIPRED PROTEIN ANALYSIS WORKBENCh: 20 years on. Nucl. Acids Res..

[24] Kumar S, Stecher G, Li M, Knyaz C, Tamura K (2018). MEGA X: molecular evolutionary genetics analysis across computing platforms. Mol. Biol. Evol..

[25] Hub JS, De Groot BL (2008). Mechanism of selectivity in aquaporins and aquaglyceroporins. PNAS.

[26] Mitani-Ueno N, Yamaji N, Zhao FJ, Ma JF (2011). The aromatic/arginine selectivity filter of NIP aquaporins plays a critical role in substrate selectivity for silicon, boron, and arsenic. J. Exp. Bot..

[27] Wallace IS, Roberts DM (2005). Distinct transport selectivity of two structural subclasses of the nodulin-like intrinsic protein family of plant aquaglyceroporin channel. Biochemistry.

[28] Dynowski M, Mayer M, Moran O, Ludewig U (2008). Molecular determinants of ammonia and urea conductance in plant aquaporin homologs. FEBS Lett..

[29] Wallace IS, Wills DM, Guenther JF, Roberts DM (2002). Functional selectivity for glycerol of the nodulin 26 subfamily of plant membrane intrinsic proteins. FEBS Lett..

[30] Jahn TP (2004). Aquaporin homologues in plants and mammals transport ammonia. FEBS Lett..

[31] Dordas C, Chrispeels MJ, Brown PH (2000). Permeability and channel-mediated transport of boric acid across membrane vesicles isolated from squash roots. Plant Physiol..

[32] Gaspar M (2003). Cloning and characterization of ZmPIP1-5b, an aquaporin transporting water and urea. Plant Sci..

[33] Liu LH, Ludewig U, Gassert B, Frommer WB, Von Wirén N (2003). Urea transport by nitrogen-regulated tonoplast intrinsic proteins in arabidopsis. Plant Physiol..

[34] Takano J (2006). The Arabidopsis major intrinsic protein NIP5;1 is essential for efficient boron uptake and plant development under boron limitation. Plant Cell.

[35] Ma JF, Yamaji N (2006). Silicon uptake and accumulation in higher plants. Trends Plant Sci..

[36] Bienert GP (2007). Specific aquaporins facilitate the diffusion of hydrogen peroxide across membranes. J. Biol. Chem..

[37] Choi WG, Roberts DM (2007). Arabidopsis NIP2;1, a major intrinsic protein transporter of lactic acid induced by anoxic stress. J. Biol. Chem..

[38] Ma JF (2008). Transporters of arsenite in rice and their role in arsenic accumulation in rice grain. Proc. Natl. Acad. Sci. USA.

[39] Mitani N, Yamaji N, Ma JF (2009). Identification of maize silicon influx transporters. Plant Cell Physiol..

[40] Quan LJ, Zhang B, Shi WW, Li HY (2008). Hydrogen peroxide in plants: a versatile molecule of the reactive oxygen species network. J. Integr. Plant Biol..

[41] Tanaka M, Wallace IS, Takano J, Roberts DM, Fujiwara T (2008). NIP6;1 is a boric acid channel for preferential transport of boron to growing shoot tissues in Arabidopsis. Plant Cell.

[42] Kamiya T (2009). NIP1;1, an aquaporin homolog, determines the arsenite sensitivity of *Arabidopsis thaliana*. J. Biol. Chem..

[43] Chiba Y, Mitani N, Yamaji N, Ma JF (2009). HvLsi1 is a silicon influx transporter in barley. Plant J..

[44] Pang Y (2010). Overexpression of the tonoplast aquaporin AtTIP5;1 conferred tolerance to boron toxicity in Arabidopsis. J. Genet. Genom..

[45] Mosa KA (2012). Members of rice plasma membrane intrinsic proteins subfamily are involved in arsenite permeability and tolerance in plants. Transgenic Res..

[46] Bárzana G, Aroca R, Bienert GP, Chaumont F, Ruiz-Lozano JM (2014). New insights into the regulation of aquaporins by the arbuscular mycorrhizal symbiosis in maize plants under drought stress and possible implications for plant performance. Mol. Plant Microbe Interact..

[47] Zhu YX (2019). Genome-wide identification, structure characterization, and expression pattern profiling of aquaporin gene family in cucumber. BMC Plant Biol..

[48] Chaumont F, Barrieu F, Wojcik E, Chrispeels MJ, Jung R (2001). Aquaporins constitute a large and highly divergent protein family in maize. Plant Physiol..

[49] Sakurai J, Ishikawa F, Yamaguchi T, Uemura M, Maeshima M (2005). Identification of 33 rice aquaporin genes and analysis of their expression and function. Plant Cell Physiol..

[50] Bussler W (2007). Mineral nutrition of plants: principles and perspectives. Z. Pflanzenernähr. Bodenkd..

[51] Hachez C, Moshelion M, Zelazny E, Cavez D, Chaumont F (2006). Localization and quantification of plasma membrane aquaporin expression in maize primary root: a clue to understanding their role as cellular plumbers. Plant Mol. Biol..

[52] Ramakers C, Ruijter JM, Lekanne Deprez RH, Moorman AFM (2003). Assumption-free analysis of quantitative real-time polymerase chain reaction (PCR) data. Neurosci. Lett..

[53] Kong Q (2014). Screening suitable reference genes for normalization in reverse transcription quantitative real-time PCR analysis in melon. PLoS ONE.

[54] Vandesompele J (2002). Accurate normalization of real-time quantitative RT-PCR data by geometric averaging of multiple internal control genes. Genome Biol..

[55] Livak KJ, Schmittgen TD (2001). Analysis of relative gene expression data using real-time quantitative PCR and the 2-ΔΔCT method. Methods.

[56] Latrasse D (2017). The quest for epigenetic regulation underlying unisexual flower development in *Cucumis melo*. Epigenet. Chromatin.

[57] Soto G, Alleva K, Amodeo G, Muschietti J, Ayub ND (2012). New insight into the evolution of aquaporins from flowering plants and vertebrates: orthologous identification and functional transfer is possible. Gene.

[58] Aharon R (2003). Overexpression of a plasma membrane aquaporin in transgenic tobacco improves plant vigor under favorable growth conditions but not under drought or salt stress. Plant Cell.

[59] Maeshima M, Ishikawa F (2008). ER membrane aquaporins in plants. Pflugers Arch. Eur. J. Physiol..

[60] Johansson I, Karlsson M, Johanson U, Larsson C, Kjellbom P (2000). The role of aquaporins in cellular and whole plant water balance. Biochim. Biophys. Acta BBA Biomembr..

[61] Yoo YJ (2016). Interactions between transmembrane helices within monomers of the aquaporin AtPIP2;1 play a crucial role in tetramer formation. Mol. Plant.

[62] Maurel C, Verdoucq L, Luu D-T, Santoni V (2008). Plant aquaporins: membrane channels with multiple integrated functions. Annu. Rev. Plant Biol..

[63] Wudick MM, Luu DT, Maurel C (2009). A look inside: localization patterns and functions of intracellular plant aquaporins. New Phytol..

[64] Soto G (2010). TIP5;1 is an aquaporin specifically targeted to pollen mitochondria and is probably involved in nitrogen remobilization in *Arabidopsis thaliana*. Plant J..

[65] Ferro M (2003). Proteomics of the chloroplast envelope membranes from Arabidopsis thaliana. Mol. Cell. Proteom..

[66] Uehlein N (2008). Function of Nicotiana tabacum aquaporins as chloroplast gas pores challenges the concept of membrane CO2 permeability. Plant Cell.

[67] Anderberg HI, Kjellbom P, Johanson U (2012). Annotation of Selaginella moellendorffli major intrinsic proteins and the evolution of the protein family in terrestrial plants. Front. Plant Sci..

[68] Wallace IS, Roberts DM (2004). Homology modeling of representative subfamilies of Arabidopsis major intrinsic proteins. Classification based on the aromatic/arginine selectivity filter. Plant Physiol..

[69] Tyerman SD, Wignes JA, Kaiser BN (2017). Root Hydraulic and Aquaporin Responses to N Availability Plant Aquaporins: From Transport to Signaling.

[70] Soto G, Alleva K, Mazzella MA, Amodeo G, Muschietti JP (2008). AtTIP1;3 and AtTIP5;1, the only highly expressed Arabidopsis pollen-specific aquaporins, transport water and urea. FEBS Lett..

[71] Beitz E, Wu B, Holm LM, Schultz JE, Zeuthen T (2006). Point mutations in the aromatic/arginine region in aquaporin 1 allow passage of urea, glycerol, ammonia, and protons. Proc. Natl. Acad. Sci. USA.

[72] Azad AK, Yoshikawa N, Ishikawa T, Sawa Y, Shibata H (2012). Substitution of a single amino acid residue in the aromatic/arginine selectivity filter alters the transport profiles of tonoplast aquaporin homologs. Biochim. Biophys. Acta Biomembr..

[73] Li GW (2008). Transport functions and expression analysis of vacuolar membrane aquaporins in response to various stresses in rice. J. Plant Physiol..

[74] Kirscht A (2016). Crystal structure of an ammonia-permeable aquaporin. PLoS Biol..

[75] Dynowski M, Schaaf G, Loque D, Moran O, Ludewig U (2008). Plant plasma membrane water channels conduct the signalling molecule H2O2. Biochem. J..

[76] Anderberg HI, Danielson JÅH, Johanson U (2011). Algal MIPs, high diversity and conserved motifs. BMC Evol. Biol..

[77] Pommerrenig B, Diehn TA, Bienert GP (2015). Metalloido-porins: Essentiality of Nodulin 26-like intrinsic proteins in metalloid transport. Plant Sci..

[78] Porquet A, Filella M (2007). Structural evidence of the similarity of Sb(OH)3 and As(OH) 3 with glycerol: implications for their uptake. Chem. Res. Toxicol..

[79] Mitani N, Yamaji N, Ma JF (2008). Characterization of substrate specificity of a rice silicon transporter, Lsi1. Pflugers Arch. Eur. J. Physiol..

[80] Kamiya T, Fujiwara T (2009). Arabidopsis NIP1;1 transports antimonite and determines antimonite sensitivity. Plant Cell Physiol..

[81] Miwa K, Fujiwara T (2010). Boron transport in plants: co-ordinated regulation of transporters. Ann. Bot..

[82] Bienert GP, Schüssler MD, Jahn TP (2008). Metalloids: essential, beneficial or toxic? Major intrinsic proteins sort it out. Trends Biochem. Sci..

[83] Li T, Choi WG, Wallace IS, Baudry J, Roberts DM (2011). Arabidopsis thaliana NIP7;1: an anther-specific boric acid transporter of the aquaporin superfamily regulated by an unusual tyrosine in helix 2 of the transport pore. Biochemistry.

[84] Ma JF, Yamaji N (2008). Functions and transport of silicon in plants. Cell. Mol. Life Sci..

[85] Deshmukh RK (2015). A precise spacing between the NPA domains of aquaporins is essential for silicon permeability in plants. Plant J..

[86] Bienert GP (2008). A subgroup of plant aquaporins facilitate the bi-directional diffusion of As(OH)3 and Sb(OH)3 across membranes. BMC Biol..

[87] Gu R, Chen X, Zhou Y, Yuan L (2012). Isolation and characterization of three maize aquaporin genes, ZmNIP2;1, ZmNIP2;4 and ZmTIP4;4 involved in urea transport. BMB Rep..

[88] Ma JF (2006). A silicon transporter in rice. Nature.

[89] Ishikawa F, Suga S, Uemura T, Sato MH, Maeshima M (2005). Novel type aquaporin SIPs are mainly localized to the ER membrane and show cell-specific expression in *Arabidopsis thaliana*. FEBS Lett..

[90] Lopez D (2012). Insights into Populus XIP aquaporins: evolutionary expansion, protein functionality, and environmental regulation. J. Exp. Bot..

[91] Maurel C, Tacnet F, Güclü J, Guern J, Ripoche P (1997). Purified vesicles of tobacco cell vacuolar and plasma membranes exhibit dramatically different water permeability and water channel activity. Proc. Natl. Acad. Sci. USA.

[92] Hachez C, Heinen RB, Draye X, Chaumont F (2008). The expression pattern of plasma membrane aquaporins in maize leaf highlights their role in hydraulic regulation. Plant Mol. Biol..

[93] Xu F (2019). Overexpression of rice aquaporin OsPIP1;2 improves yield by enhancing mesophyll CO2 conductance and phloem sucrose transport. J. Exp. Bot..

[94] Bienert GP, Chaumont F (2014). Aquaporin-facilitated transmembrane diffusion of hydrogen peroxide. Biochem. Biophys. Acta.

[95] Jang JY, Kim DG, Kim YO, Kim JS, Kang H (2004). An expression analysis of a gene family encoding plasma membrane aquaporins in response to abiotic stresses in *Arabidopsis thaliana*. Plant Mol. Biol..

[96] Zhang Y, Wang Z, Chai T, Wen Z, Zhang H (2008). Indian mustard aquaporin improves drought and heavy-metal resistance in tobacco. Mol. Biotechnol..

[97] Heinen RB, Ye Q, Chaumont F (2009). Role of aquaporins in leaf physiology. J. Exp. Bot..

[98] Fetter K, Van Wilder V, Moshelion M, Chaumont F (2004). Interactions between plasma membrane aquaporins modulate their water channel activity. Plant Cell.

[99] Zelazny E (2007). FRET imaging in living maize cells reveals that plasma membrane aquaporins interact to regulate their subcellular localization. Proc. Natl. Acad. Sci. USA.

[100] Ma JF, Yamaji N (2015). A cooperative system of silicon transport in plants. Trends Plant Sci..

[101] Bustin SA (2002). Quantification of mRNA using real-time reverse transcription PCR (RT-PCR): trends and problems. J. Mol. Endocrinol..

[102] Derveaux S, Vandesompele J, Hellemans J (2010). How to do successful gene expression analysis using real-time PCR. Methods.

[103] Vandeleur RK (2009). The role of plasma membrane intrinsic protein aquaporins in water transport through roots: diurnal and drought stress responses reveal different strategies between isohydric and anisohydric cultivars of grapevine. Plant Physiol..

