# Supplementary material for: Genome-wide analysis of the aquaporin genes in melon (Cucumis melo L.)
Source: Sci Rep. 2020 Dec 17;10:22240. doi: 10.1038/s41598-020-79250-w (PMC7747737; doi:10.1038/s41598-020-79250-w)
Supplement: Supplementary file 1 — Supplementary Information. [file 41598_2020_79250_MOESM1_ESM.pdf]

Genome-Wide Analysis of the Aquaporin Genes in Melon (*Cucumis melo L.*)

Alvaro Lopez-Zaplana, Juan Nicolas-Espinosa, Micaela Carvajal and Gloria Bárzana*

Supplementary Figure S1. Exon-intron structures of all *Cucumis melo* AQPs using the data available in NCBI data base (https://www.ncbi.nlm.nih.gov/). Analysis of sequences were performed using Microsoft Office package (2016) software.

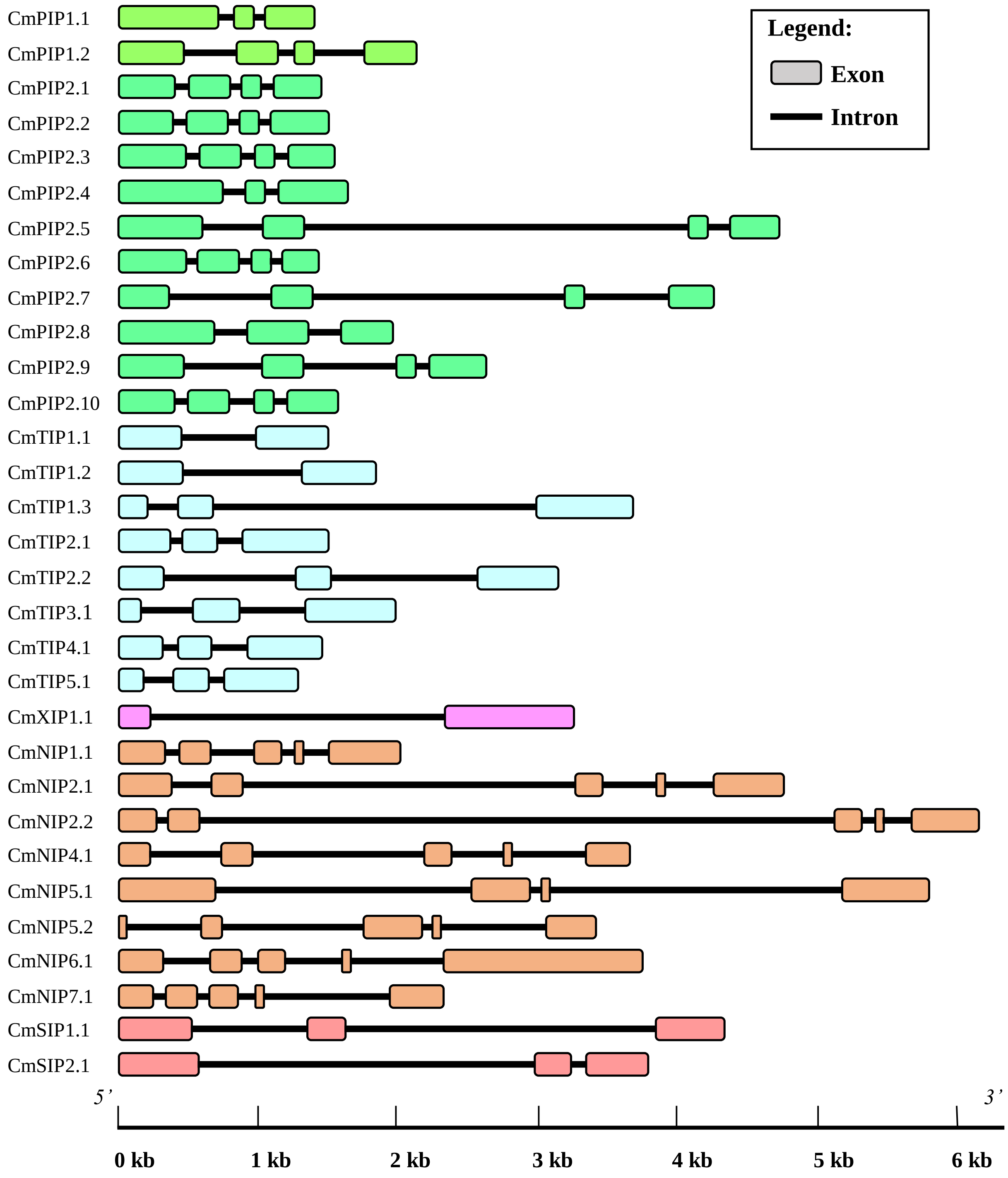

Aquaporins Group. Plant Nutrition Department, Centro de Edafología y Biología Aplicada del Segura (CEBAS-CSIC), Campus Universitario de Espinardo, Edificio 25, 30100 Murcia, Spain.
*Corresponding author: Gloria Bárzana González. e-mail: gbarzana@cebas.csic.es

Supplementary Figure S2. Phylogenetic analysis of AQP family proteins of *Cucumis melo L.* (Cm), *Arabidopsis thaliana L.* (At), *Zea mays L.* (Zm) and *Oryza sativa L.* (Os). MUSLE was used to align the protein sequences and the NJ method (with 1000 bootstrap replications) to build the tree, all with MEGA X. Phylogenetic tree design has been done with the online tool "Interactive Tree Of Life" (iTOL; https://itol.embl.de/).

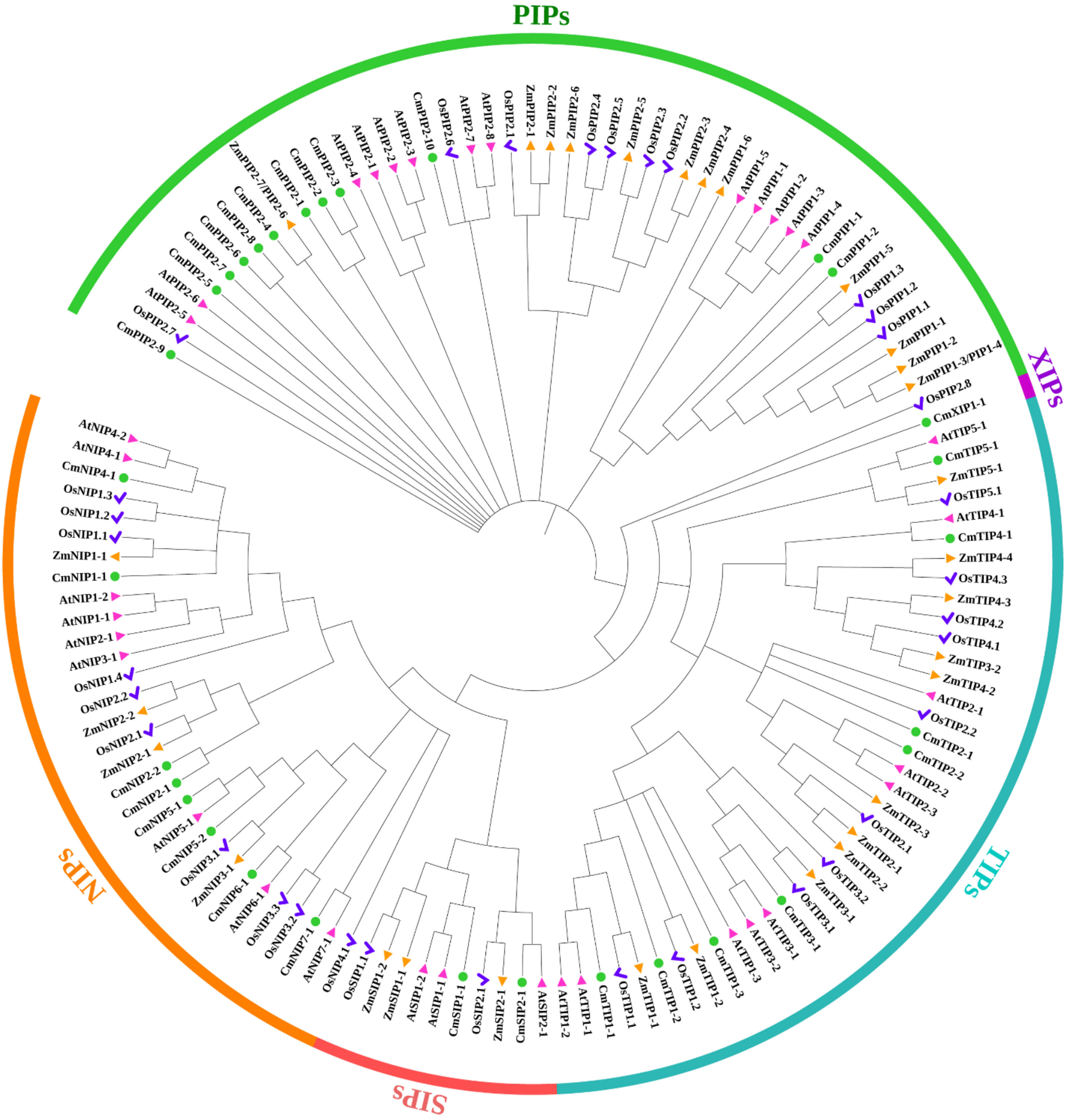

Aquaporins Group. Plant Nutrition Department, Centro de Edafología y Biología Aplicada del Segura (CEBAS-CSIC), Campus Universitario de Espinardo, Edificio 25, 30100 Murcia, Spain.
*Corresponding author: Gloria Bárzana González. e-mail: gbarzana@cebas.csic.es

Supplementary Figure S3. Motif locations was performed by MEME web server (http://meme-suite.org/tools/meme) using the complete protein sequences of all *Cucumis melo* AQPs using the data available in NCBI data base. 15 conserved motifs were selected using different colored boxes.

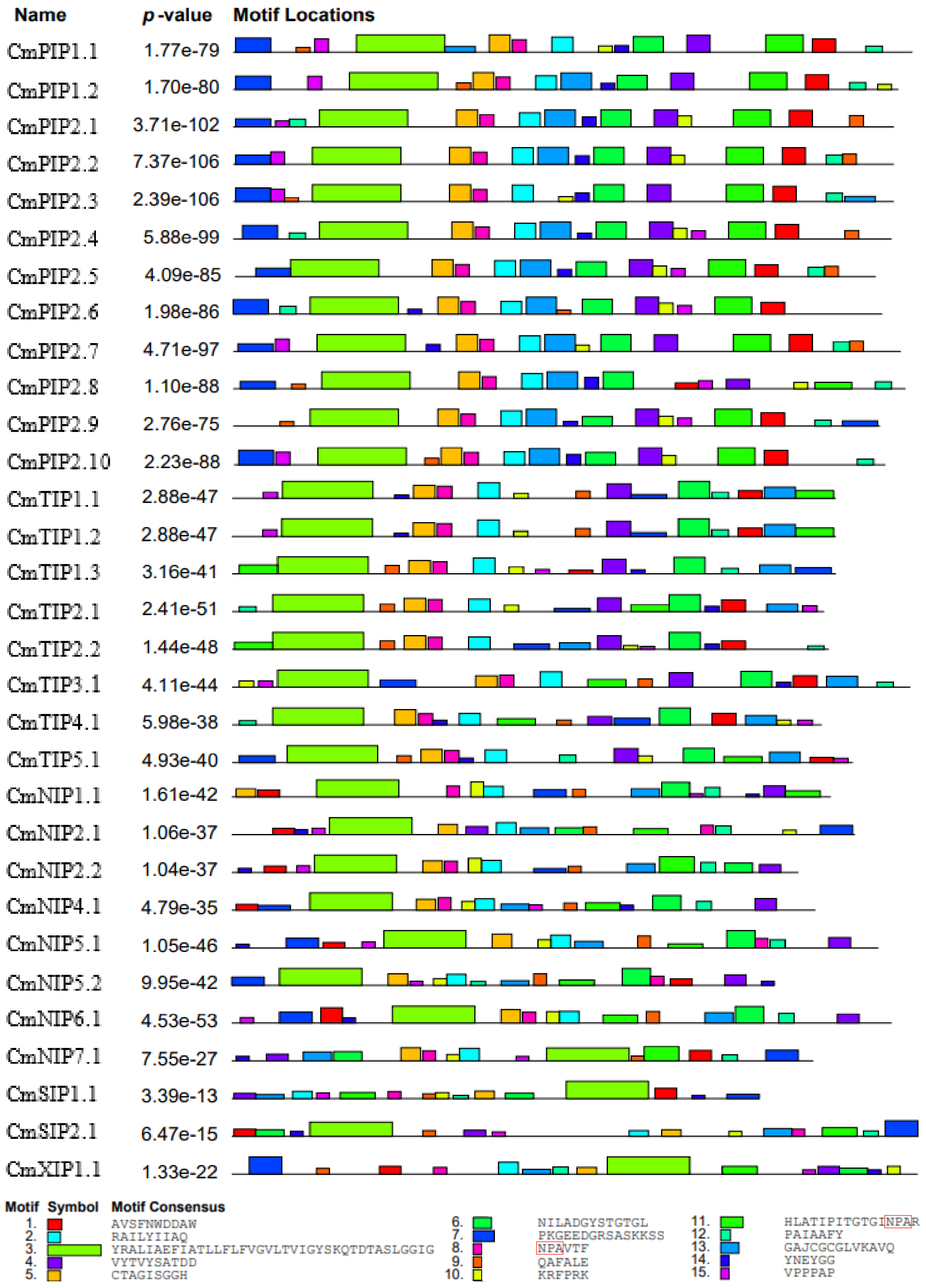

Aquaporins Group. Plant Nutrition Department, Centro de Edafología y Biología Aplicada del Segura (CEBAS-CSIC), Campus Universitario de Espinardo, Edificio 25, 30100 Murcia, Spain.
*Corresponding author: Gloria Bárzana González. e-mail: gbarzana@cebas.csic.es

Supplementary Table S1. Identification of important residues for *Arabidopsis thaliana L.* (At), *Zea mais L.* (Zm) and *Oryza sativa L.* (Os) aquaporins. Columns: Plant species (PS); protein name; NPA motif (LB and LE positions); ar/R selectivity filter (H2, H5, LE1and LE2 positions) and Froger's positions (P1-P5) a) Tonoplast intrinsic proteins (TIPs) b) Plasma membrane intrinsic proteins (PIPs) c) Nodulin26-like intrinsic proteins (NIPs) and small basic intrinsic proteins (SIPs).

a) Table of important positions for tonoplast intrinsic proteins (TIPs)

PS	Protein name	NPA motif	ar/R selectivity filter				Froger's positions				
		LB/LE	H2	H5	LE1	LE2	P1	P2	P3	P4	P5
Arabidopsis thaliana L.	AtTIP1.1	NPA/NPA	H	I	A	V	T	A	A	Y	W
	AtTIP1.2	NPA/NPA	H	I	A	V	T	A	A	Y	W
	AtTIP1.3	NPA/NPA	H	I	A	V	T	S	A	Y	W
	AtTIP2.1	NPA/NPA	H	I	G	R	T	S	A	Y	W
	AtTIP2.2	NPA/NPA	H	I	G	R	T	S	A	Y	W
	AtTIP2.3	NPA/NPA	H	I	G	R	T	S	A	Y	W
	AtTIP3.1	NPA/NPA	H	I	A	R	T	A	A	Y	W
	AtTIP3.2	NPA/NPA	H	I	A	R	T	A	A	Y	W
	AtTIP4.1	NPA/NPA	H	I	A	R	T	S	A	Y	W
	AtTIP5.1	NPA/NPA	N	V	G	C	V	A	A	Y	W
Zea mais L.	OsTIP1.1	NPA/NPA	H	I	A	V	T	S	A	Y	W
	OsTIP1.2	NPA/NPA	H	I	A	V	T	S	A	Y	W
	OsTIP2.1	NPA/NPA	H	I	G	R	T	S	A	Y	W
	OsTIP2.2	NPA/NPA	H	I	G	R	T	S	A	Y	W
	OsTIP2.3	NPA/NPA	H	I	S	R	T	S	A	Y	W
	OsTIP3.1	NPA/NPA	H	M	A	R	T	V	A	Y	W
	OsTIP3.2	NPA/NPA	H	I	A	R	S	A	A	Y	W
	OsTIP4.1	NPA/NPA	T	T	A	R	G	S	A	Y	W
	OsTIP4.2	NPA/NPA	Q	T	A	R	G	S	A	Y	W
	OsTIP4.3	NPA/NPA	H	I	A	R	G	S	A	Y	W
	OsTIP5.1	NPA/NPA	Q	V	A	R	A	S	A	Y	W
Oryza sativa L.	ZmTIP1.1	NPA/NPA	H	I	A	V	T	S	A	Y	W
	ZmTIP1.2	NPA/NPA	H	I	A	V	T	S	A	Y	W
	ZmTIP2.1	NPA/NPA	H	I	G	R	T	S	A	Y	W
	ZmTIP2.2	NPA/NPA	H	I	G	R	T	S	A	Y	W
	ZmTIP2.3	NPA/NPA	H	I	G	R	T	S	A	Y	W
	ZmTIP3.1	NPA/NPA	H	V	A	R	T	V	A	Y	W
	ZmTIP3.2	NPA/NPA	H	V	A	R	T	V	A	Y	W
	ZmTIP4.1	NPA/NPA	H	S	A	R	S	S	A	Y	W
	ZmTIP4.2	NPA/NPA	H	S	A	R	S	S	A	Y	W
	ZmTIP4.3	NPA/NPA	Q	S	A	R	T	S	A	Y	W
	ZmTIP4.4	NPA/NPA	H	V	A	R	A	S	A	Y	W
	ZmTIP5.1	NPA/NPA	Q	V	A	R	S	S	A	Y	W

Shortening codes: LB, loop B; LE, loop E; H, hélix.
Aminoacide residues named with letters according the international code.

Aquaporins Group. Plant Nutrition Department, Centro de Edafología y Biología Aplicada del Segura (CEBAS-CSIC), Campus Universitario de Espinardo, Edificio 25, 30100 Murcia, Spain.
*Corresponding author: Gloria Bárzana González. e-mail: gbarzana@cebas.csic.es

b) Table of important positions for plasma membrane intrinsic proteins (PIPs)

PS	Protein name	NPA motif	ar/R selectivity filter				Froger's positions				
		LB/LE	H2	H5	LE1	LE2	P1	P2	P3	P4	P5
Arabidopsis thaliana L.	AtPIP1;1	NPA/NPA	F	H	T	R	Q	S	A	F	W
	AtPIP1;2	NPA/NPA	F	H	T	R	Q	S	A	F	W
	AtPIP1;3	NPA/NPA	F	H	T	R	Q	S	A	F	W
	AtPIP1;4	NPA/NPA	F	H	T	R	Q	S	A	F	W
	AtPIP1;5	NPA/NPA	F	H	T	R	Q	S	A	F	W
	AtPIP2;1	NPA/NPA	F	H	T	R	Q	S	A	F	W
	AtPIP2;2	NPA/NPA	F	H	T	R	Q	S	A	F	W
	AtPIP2;3	NPA/NPA	F	H	T	R	Q	S	A	F	W
	AtPIP2;4	NPA/NPA	F	H	T	R	Q	S	A	F	W
	AtPIP2;5	NPA/NPA	F	H	T	R	Q	S	A	F	W
	AtPIP2;6	NPA/NPA	F	H	T	R	Q	S	A	F	W
	AtPIP2;7	NPA/NPA	F	H	T	R	M	S	A	F	W
	AtPIP2;8	NPA/NPA	F	H	T	R	M	S	A	F	W
Zea mais L.	ZmPIP1;1	NPA/NPA	F	H	T	R	Q	S	A	F	W
	ZmPIP1;2	NPA/NPA	F	H	T	R	Q	S	A	F	W
	ZmPIP1;3	NPA/NPA	F	H	T	R	Q	S	A	F	W
	ZmPIP1;4	NPA/NPA	F	H	T	R	Q	S	A	F	W
	ZmPIP1;5	NPA/NPA	F	H	T	R	Q	S	A	F	W
	ZmPIP1;6	NPA/NPA	F	H	T	R	G	S	A	F	W
	ZmPIP2;1	NPA/NPA	F	H	T	R	Q	S	A	F	W
	ZmPIP2;2	NPA/NPA	F	H	T	R	Q	S	A	F	W
	ZmPIP2;3	NPA/NPA	F	H	T	R	Q	S	A	F	W
	ZmPIP2;4	NPA/NPA	F	H	T	R	Q	S	A	F	W
	ZmPIP2;5	NPA/NPA	F	H	T	R	Q	S	A	F	W
	ZmPIP2;6	NPA/NPA	F	H	T	R	Q	S	A	F	W
	ZmPIP2;7	NPA/NPA	F	H	T	R	Q	S	A	F	W
Oryza sativa L.	OsPIP1;1	NPA/NPA	F	H	T	R	Q	S	A	F	W
	OsPIP1;2	NPA/NPA	F	H	T	R	Q	S	A	F	W
	OsPIP1;3	NPA/NPA	F	H	T	R	Q	S	A	F	W
	OsPIP2;1	NPA/NPA	F	H	T	R	Q	S	A	F	W
	OsPIP2;2	NPA/NPA	F	H	T	R	Q	S	A	F	W
	OsPIP2;3	NPA/NPA	F	H	T	R	Q	S	A	F	W
	OsPIP2;4	NPA/NPA	F	H	T	R	Q	S	A	F	W
	OsPIP2;5	NPA/NPA	F	H	T	R	Q	S	A	F	W
	OsPIP2;6	NPA/NPA	F	H	T	R	M	S	A	F	W
	OsPIP2;7	NPA/NPA	F	H	T	R	M	S	A	F	W
	OsPIP2;8	NPA/NPA	F	H	T	R	M	S	A	F	W

Shortening codes: LB, loop B; LE, loop E; H, hélix.
Aminoacide residues named with letters according the international code.

Aquaporins Group. Plant Nutrition Department, Centro de Edafología y Biología Aplicada del Segura (CEBAS-CSIC), Campus Universitario de Espinardo, Edificio 25, 30100 Murcia, Spain.
*Corresponding author: Gloria Bárzana González. e-mail: gbarzana@cebas.csic.es

c) Table of important positions for nodulin-like intrinsic proteins (NIPs) and small basic intrinsic proteins (SIPs).

PS	Protein name	NPA motif	ar/R selectivity filter				Froger's positions				
		LB/LE	H2	H5	LE1	LE2	P1	P2	P3	P4	P5
Arabidopsis thaliana L.	AtNIP1.1	NPA/NPA	W	V	A	R	F	S	A	Y	L
	AtNIP1.2	NPA/NPA	W	V	A	R	F	S	A	Y	I
	AtNIP2.1	NPA/NPA	W	V	A	R	F	S	A	Y	L
	AtNIP3.1	NPA/NPA	W	I	A	R	F	S	A	Y	I
	AtNIP4.1	NPA/NPA	W	V	A	R	F	S	A	Y	I
	AtNIP4.2	NPA/NPA	W	V	A	R	F	S	A	Y	I
	AtNIP5.1	NPS/NPV	A	I	G	R	F	T	A	Y	L
	AtNIP6.1	NPA/NPV	A	I	A	R	F	T	A	Y	L
	AtNIP7.1	NPS/NPA	A	V	G	R	Y	S	A	Y	M
	AtSIP1.1	NPT/NPA	I	V	P	I	I	A	A	Y	W
	AtSIP1.2	NPC/NPA	V	F	P	I	I	A	A	Y	W
	AtSIP2.1	NPL/NPA	S	H	G	A	F	V	A	Y	W
Oryza sativa L.	OsNIP1.1	NPA/NPA	W	V	A	R	F	S	A	Y	I
	OsNIP1.2	NPA/NPA	W	V	A	R	F	S	A	Y	V
	OsNIP1.3	NPA/NPA	W	V	A	R	F	T	A	Y	I
	OsNIP1.4	NPA/NPA	W	V	A	R	F	S	A	Y	I
	OsNIP1.5	NPA/NPA	W	V	A	R	F	S	A	Y	V
	OsNIP2.1	NPA/NPA	G	S	G	R	I	T	A	Y	F
	OsNIP2.2	NPA/NPA	G	S	G	R	L	T	A	Y	F
	OsNIP3.1	NPS/NPV	A	I	G	R	F	T	A	Y	L
	OsNIP3.2	NPA/NPA	A	A	A	R	Y	T	A	Y	L
	OsNIP3.3	NPA/NPA	A	I	A	R	Y	T	A	Y	L
	OsNIP3.4	NPA/NPA	I	V	A	R	F	S	A	Y	M
	OsNIP3.5	NPA/NPA	A	A	P	R	Y	T	A	Y	L
	OsSIP1.1	NPT/NPA	L	V	P	N	M	A	A	Y	W
	OsSIP2.1	NPL/NPA	S	H	G	S	L	A	A	Y	W
Zea mais L.	ZmNIP1.1	NPA/NPA	W	V	A	R	F	S	A	Y	V
	ZmNIP2.1	NPA/NPA	G	S	G	R	L	T	A	Y	F
	ZmNIP2.2	NPA/NPA	G	S	G	R	L	T	A	Y	F
	ZmNIP2.3	NPA/NPA	G	S	G	R	L	T	A	Y	F
	ZmNIP3.1	NPS/NPV	A	I	G	R	F	T	A	Y	L
	ZmSIP1.1	NPT/NPA	L	I	P	N	M	A	A	Y	W
	ZmSIP1.2	NPT/NPA	L	V	P	N	M	A	A	Y	W
	ZmSIP2.1	NPL/NPA	S	H	G	S	F	A	A	Y	W

Shortening codes: LB, loop B; LE, loop E; H, hélix.
Aminoacide residues named with letters according the international code.

Aquaporins Group. Plant Nutrition Department, Centro de Edafología y Biología Aplicada del Segura (CEBAS-CSIC), Campus Universitario de Espinardo, Edificio 25, 30100 Murcia, Spain.
*Corresponding author: Gloria Bárzana González. e-mail: gbarzana@cebas.csic.es
